# Effects of the Combined Incorporation of ZnO and TiO_2_ Nanoparticles on the Mechanical, Rheological, Thermal, and Healing Properties of a Dense Polymeric Asphalt Mixture

**DOI:** 10.3390/nano15231779

**Published:** 2025-11-26

**Authors:** Jaqueline Wolfart, João Victor Staub de Melo, Alexandre Luiz Manfro, Breno Salgado Barra, Rafael Cassimiro Barbosa

**Affiliations:** Department of Civil Engineering, Federal University of Santa Catarina, Rua João Pio Duarte Silva, Florianópolis 88040-970, SC, Brazil

**Keywords:** zinc oxide, titanium dioxide, polymeric asphalt binder, nanoparticles, asphalt nanocomposites, asphalt mixtures, mechanical behavior, healing

## Abstract

This study evaluated the combined incorporation of zinc oxide (ZnO) and titanium dioxide (TiO_2_) nanoparticles into a styrene–butadiene–styrene (SBS) copolymer-modified asphalt binder, aiming to increase thermal conductivity and healing potential while maintaining rheological performance. Nanocomposites containing ZnO + TiO_2_ (50/50 wt.%) were produced at dosages of 2–12 wt.% and subjected to the Rolling Thin Film Oven Test (RTFOT), thermal conductivity measurements, viscosity testing, and rheological characterization. A dense-graded asphalt mixture with the optimized dosage was evaluated through wheel-tracking, four-point bending fatigue and healing, and internal heating rate assessment under microwave radiation. The integrated results indicated 8.5 wt.% as the optimal dosage, providing a 106.3% increase in thermal conductivity and improving the high-temperature performance grade (PGH) from 76-XX to 82-XX. Non-recoverable creep compliance (Jnr) decreased by 21.1%, and viscosity at 135 °C increased by 41.8%, remaining below 3.0 Pa·s. In the asphalt mixture, healing capacity increased by 50.7%, and the internal heating rate by 50.0%, while the wheel-tracking rut depth decreased by 13.3%. These findings demonstrate that 8.5 wt.% ZnO + TiO_2_ simultaneously enhances heat conduction, healing efficiency, and resistance to permanent deformation, offering a promising solution for pavements subjected to high temperatures and heavy traffic.

## 1. Introduction

Metallic semiconductor nanomaterials, such as zinc oxide (ZnO) and titanium dioxide (TiO_2_), have been widely investigated due to their photocatalytic properties, particularly their ability to degrade environmental pollutants [1,2,3,4]. In pavement engineering, these materials have been incorporated into cementitious [5,6] and asphalt matrices [7,8,9,10] with the aim of reducing pollutant concentrations in urban environments. In asphalt binders and mixtures, typical dosages range from 2 to 15 wt.% [7,8,10,11,12,13].

Beyond their photocatalytic activity, the isolated use of nano-ZnO and nano-TiO_2_ in asphalt binders has been extensively studied for their effects on high-temperature rheological performance. Studies show that increasing the concentration of these nanomaterials raises the apparent viscosity [14,15], the high-temperature performance grade (PGH) [7,14,16], and the dynamic shear modulus (|G*|) [7,14,17,18,19], while reducing the non-recoverable creep compliance (Jnr) [7,14,20] and the phase angle (δ) [17,18,19], consistently indicating improvements in resistance to permanent deformation.

At the mixture scale, the incorporation of TiO_2_ has shown significant gains in indirect tensile strength, stiffness modulus, fracture energy, and rutting resistance [15,21,22]. Similarly, ZnO has been reported to enhance crack propagation resistance, fracture energy, moisture resistance, and permanent deformation performance [23,24,25].

Despite these advances, the literature predominantly investigates ZnO and TiO_2_ independently, and knowledge of their combined effects remains limited, particularly in SBS-modified systems. Recent studies have used both oxides, but with a primarily physicochemical or binder-level rheological focus. For example, Fu et al. [26] studied composite TiO_2_/ZnO–basalt fiber-modified binders, evaluating microstructure and rheology. Xie et al. [27] investigated an SBS/ZnO/TiO_2_ system aimed at improving UV resistance and low-temperature behavior through physico-mechanical testing and aging indices. Rocha Segundo et al. [28] combined low dosages of TiO_2_ and ZnO to impart photocatalytic activity to the binder, evaluating physicochemical behavior and rheology after aging. Zhang et al. [29] employed combinations of ZnO, TiO_2_, and polymers to simultaneously improve high- and low-temperature performance of base asphalts.

However, none of these studies evaluated ZnO + TiO_2_ nanocomposites in SBS-modified binders using methodologies oriented toward the functional performance of asphalt mixtures, such as four-point bending fatigue, intermediate-temperature rheology, microwave-induced internal heating, healing, or wheel-tracking permanent deformation. Thus, the integrated application of these performance-related analyses to such nanocomposites remains unexplored in the literature.

Another relevant gap concerns the behavior of polymeric asphalt binders. Although SBS improves resistance to permanent deformation and viscoelastic performance, its intrinsic healing capacity is limited because the molecular diffusion required for microcrack closure is often insufficient due to restricted internal heat redistribution. Considering the metallic and semiconducting nature of ZnO and TiO_2_ [30,31,32], as well as their thermal conductivity [33,34], it is hypothesized that these nanomaterials may enhance heat conduction and redistribution, promoting more favorable healing conditions in SBS binders. Furthermore, their role as physical reinforcement may contribute to increased structural stiffness, more uniform thermal response, and improved polymer dispersion, supporting enhanced performance under severe conditions [35,36].

Given this scenario, this study presents the first comprehensive evaluation of ZnO + TiO_2_ nanocomposites applied to SBS-modified binders, establishing an optimal dosage based on combined thermal and rheological criteria and demonstrating their multiscale effects on asphalt mixtures. The investigation examined whether dosages commonly used in photocatalytic applications could also enhance the healing potential of dense asphalt mixtures, thereby expanding the functional role of these nanomaterials beyond photocatalysis. This approach sought to develop a mixture capable not only of achieving high mechanical performance at elevated temperatures but also of leveraging these conditions to intensify the healing process, which represents an important strategy to mitigate failures such as rutting, particularly in scenarios where thermal healing and photocatalytic activity are most effective.

For this purpose, ZnO and TiO_2_ were incorporated at a 1:1 ratio (50/50 wt.%) and at concentrations ranging from 2 to 12 wt.% (in 2% increments) in a polymeric asphalt binder. Thermal conductivity and rheological tests were used to map the dose–response behavior, identify inflection points between reinforcement and potential penalties, and determine the optimal dosage range. This optimal dosage was then applied to mixtures subjected to wheel-tracking tests, intermediate-temperature rheology, fatigue, healing, and microwave-induced heating, providing quantitative evidence of the effectiveness of the ZnO + TiO_2_ combination for high-performance pavement applications.

## 2. Materials

The materials used in this investigation consisted of crushed mineral aggregates, a polymer-modified asphalt binder, and metallic oxide nanoparticles (ZnO and TiO_2_).

The crushed mineral aggregates were of granitic origin and were supplied by Sul Brasil Mineração (SBM), located in the municipality of Paulo Lopes, in the state of Santa Catarina, Brazil. These aggregates were selected for their suitable properties for use in asphalt mixtures, meeting the required specifications. Table 1 presents the main physical and mechanical characteristics of the aggregates, which are essential for asphalt mixture design.

The polymeric asphalt binder used in this research was manufactured and supplied by CBB Asfaltos, located in the municipality of Curitiba, in the state of Paraná, Brazil. It consists of a binder modified with 4% of a styrene-butadiene-styrene (SBS) copolymer, specifically the D1101 A grade, commercialized by Kraton Polymers (Houston, TX, USA). The modification was carried out on an industrial scale, ensuring homogeneity and quality of the final product. The main properties of the polymeric asphalt binder are presented in Table 2, while the technical characteristics of the incorporated SBS copolymer are detailed in Table 3.

The ZnO and TiO_2_ nanoparticles, both with dimensions in the nanometric range, were purchased from Nanostructured & Amorphous Materials, Inc., located in Houston, TX, USA. These nanomaterials were selected due to their specific physicochemical properties, such as high surface area, high purity, and controlled crystalline structure. Table 4 presents the main technical characteristics provided by the manufacturer. In addition to the information supplied by the manufacturer, field emission scanning electron microscopy (FEG-SEM) and thermal analyses, including thermogravimetry (TGA) and derivative thermogravimetry (DTG), were performed.

**Figure 1:**
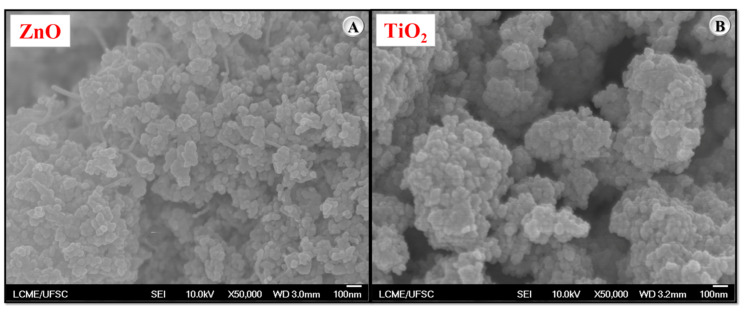
Scanning electron micrographs of (**A**) zinc oxide (ZnO) and (**B**) titanium dioxide (TiO_2_).

The morphological characterization of the nanoparticles was carried out using field emission scanning electron microscopy (FEG-SEM) to assess particle shape and size at the nanometric scale. The tests were conducted with an ultra–high-vacuum field emission scanning electron microscope, model JEOL JSM-6701F (Akishima, Tokyo, Japan). To obtain high-resolution and high-quality images, consistent operational parameters were adopted, including a magnification of 50,000× and an acceleration voltage of 10 kV. The resulting micrographs (Figure 1) reveal the morphological features of the ZnO and TiO_2_ nanoparticles.

Figure 1 shows the scanning electron micrographs of zinc oxide (ZnO) (Figure 1A) and titanium dioxide (TiO_2_) (Figure 1B) nanoparticles. Both materials exhibit predominantly spherical to ellipsoidal morphology. Additionally, both types of nanoparticles have dimensions below 100 nm, as evidenced by the micrographs, which is consistent with the characteristics reported by the manufacturer.

**Figure 2:**
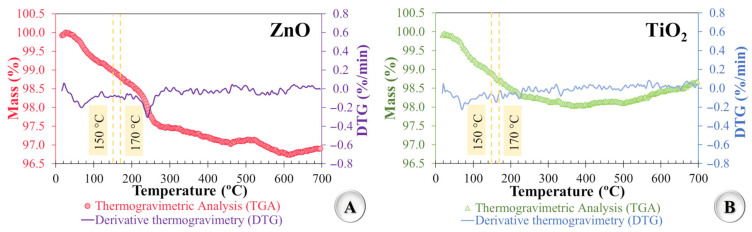
Thermogravimetric curves of (**A**) zinc oxide (ZnO) and (**B**) titanium dioxide (TiO_2_) nanoparticles.

The thermogravimetric (TGA) and derivative thermogravimetric (DTG) analyses were conducted to assess the thermal stability of the nanoparticles, considering the thermal environment typical of asphalt mixture production and application stages. The analyses were performed using an STA 449 F3 Jupiter^®^—Netzsch (Selb, Bavaria, Germany) thermoanalyzer, following the procedures described in ASTM E2550 [61]. The mass loss curves as a function of temperature for both nanomaterials are presented in Figure 2. In the presented graphs, particular emphasis was given to the temperature range between 150 °C and 170 °C, since this interval corresponds to the thermal conditions commonly used during polymeric asphalt binder modification and asphalt mixture production and compaction. The evaluation of thermogravimetric behavior within this range is crucial, as the thermal stability of the nanomaterials determines their ability to withstand degradation or significant mass loss during processing. Such analysis ensures that the nanomaterials retain their functional properties and contribute effectively to the performance of the asphalt mixture throughout the entire production and application process.

As shown in Figure 2A, ZnO exhibited a mass loss of 1.0% at 150 °C and 1.2% at 170 °C. Similarly, Figure 2B shows that TiO_2_ exhibited a mass loss of 1.1% at 150 °C and 1.3% at 170 °C. These slight mass reductions, observed within the temperature range typical of processes involving asphalt mixtures, are exclusively associated with particle dehydration, that is, the release of physically adsorbed water from the surface, and not with chemical or structural degradation of the materials [62,63,64,65].Therefore, the results confirm the adequate thermal stability of the nanomaterials (ZnO and TiO_2_) within the operational temperature range relevant to asphalt paving applications.

In summary, the selected materials (granitic aggregates, polymeric asphalt binder with SBS D1101 A (Kraton Polymers, Houston, TX, USA), and ZnO and TiO_2_ nanoparticles) provide the foundation for the experimental program. The following sections describe the procedures for binder nanomodification, thermal and rheological evaluation for determining the optimal nanoparticle content, and, finally, the mechanical testing of the asphalt mixtures.

## 3. Methods

**Figure 3:**
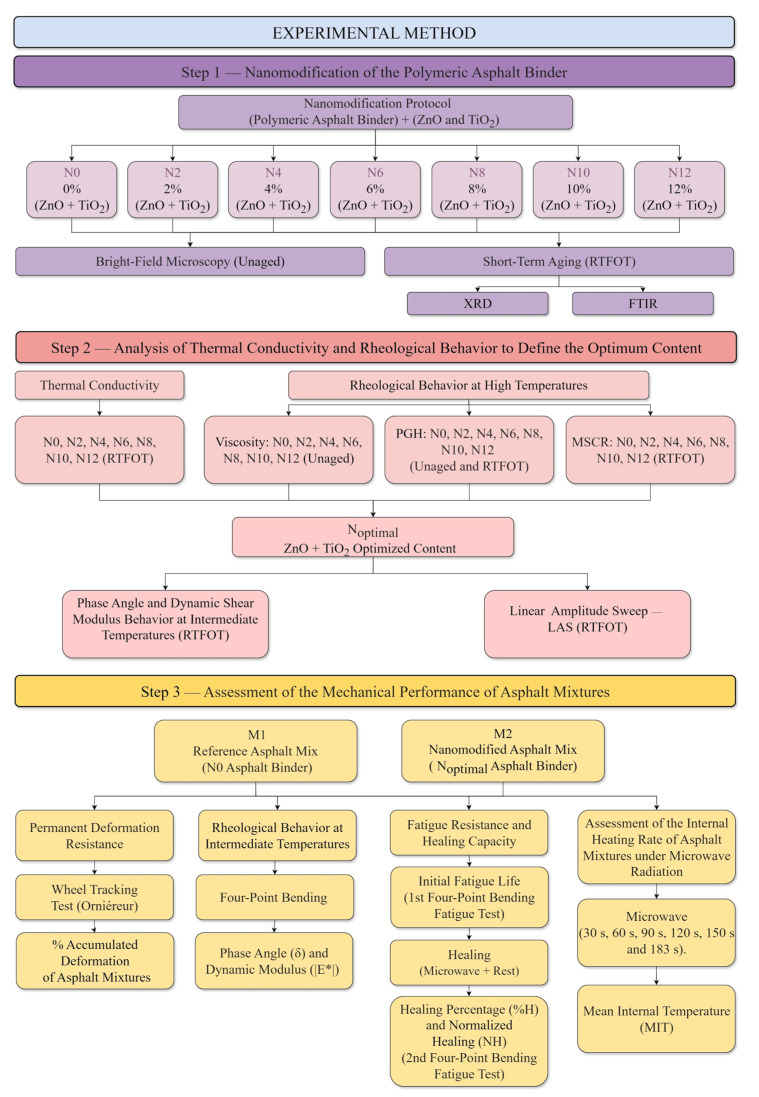
Diagram of the experimental method.

To achieve the objectives proposed in this research, an experimental program was structured in sequential and interdependent stages. The principal phases that comprised the experimental development are presented below: (Step 1) Nanomodification of the Polymeric Asphalt Binder; (Step 2) Analysis of Thermal Conductivity and Rheological Behavior to Define the Optimum Content; and (Step 3) Assessment of the Mechanical Performance of Asphalt Mixtures. The overall structure of the adopted methodology is presented schematically in Figure 3. In the subsequent sections, each stage composing the experimental procedure is described in detail to ensure the reproducibility and understanding of the investigative process employed.

### 3.1. Nanomodification of the Polymeric Asphalt Binder

The nanomodification of the polymeric asphalt binder was carried out by incorporating combined concentrations of ZnO and TiO_2_ ranging from 2%, 4%, 6%, 8%, 10%, to 12% by weight. The selection of this concentration range (2–12 wt.%, in 2% increments) was based on literature references that investigated the isolated use of these nanomaterials in studies focused on the photocatalytic degradation of pollutants in asphalt matrices [7,8,10,11,12,13]. Establishing this interval enabled the assessment of whether concentrations typically employed in photocatalytic applications could also enhance the self-healing potential of a dense asphalt mixture, thereby extending the functional performance of the nanomaterials beyond their conventional photocatalytic role.

A 1:1 mass ratio (50/50 wt.%) between ZnO and TiO_2_ was maintained across all addition levels. This proportion was adopted based on studies conducted in non-asphaltic matrices [66,67,68], which indicate that equimass ZnO–TiO_2_ formulations tend to exhibit superior photocatalytic performance compared with asymmetric ratios. It is also noteworthy that studies evaluating the combined use of ZnO and TiO_2_ in asphalt binders or mixtures remain scarce, which further justified the adoption of the 1:1 ratio as the reference condition in this research. The detailed composition corresponding to each concentration level used in this phase of the study is presented in Table 5.

**Figure 4:**
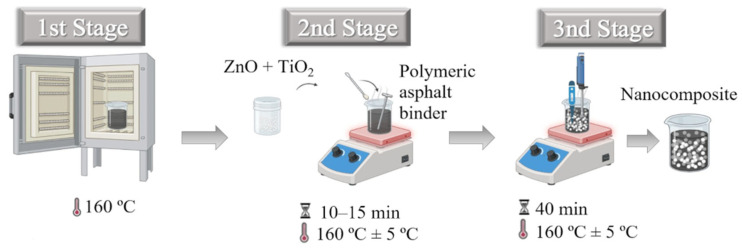
Nanomodification protocol of polymeric reference asphalt binder.

The nanomodification protocol for the polymeric asphalt binder, established based on previous studies conducted by the research group involving nanoparticle-modified binders [35,36,69], was designed to ensure methodological consistency and direct comparability with those works. The procedure was structured into three main stages (Figure 4). In the first stage, the binder samples were preheated in an oven at 160 ± 5 °C (apparent viscosity between 0.510 and 0.777 Pa·s), a temperature widely employed in the literature for the nanomodification of asphalt binders [7,26,28,70]. In the second stage, the nanomaterials were manually added to approximately 200 mL of base binder under continuous stirring for 10 to 15 min, ensuring their initial wetting and pre-dispersion. In the third stage, high-shear mixing was performed using a 700 W Philco^®^ electric mixer (Joinville, Santa Catarina, Brazil) operating at 6000 rpm. The process lasted 40 min, conducted in 1 min stirring cycles followed by 1 min of rest, resulting in 20 min of effective high-shear application. The total energy density applied to the system during the nanomodification process was 404.5 J/m^3^.

#### 3.1.1. Bright-Field Microscopy

After the nanomodification process, bright-field microscopy was performed to investigate the distribution of the nanomaterials within the asphalt binder. Micrographs were obtained using an Olympus IX83 inverted microscope (Shinjuku, Tokyo, Japan), operating under diascopic bright-field illumination and equipped with a 40× objective lens. The unaged samples N0, N2, N4, N6, N8, N10, and N12 were analyzed. Sample preparation consisted of depositing and spreading a small amount of asphalt binder onto 26 mm × 76 mm glass slides to form a thin, homogeneous film. The samples were then covered with two 20 mm × 20 mm glass coverslips, each with a controlled thickness of 0.13 mm and 0.16 mm, to protect the material and enable the acquisition of higher-resolution images.

#### 3.1.2. Short-Term Aging

Following the experimental procedure, short-term aging of the binder samples was performed using the Rotary Thin Film Oven Test (RTFOT) to simulate the thermal and oxidative conditions to which asphalt matrices are subjected during production and paving operations. The residues obtained after RTFOT were subsequently used for characterization and performance testing, including X-ray diffraction (XRD), Fourier-transform infrared spectroscopy (FTIR), thermal conductivity measurement, high-temperature performance grade (PGH) evaluation, assessment of susceptibility to permanent deformation, phase angle and dynamic shear modulus behavior at intermediate temperatures, as well as the linear amplitude sweep (LAS) test.

Short-term aging was carried out in accordance with ASTM D2872 [71], using a Rolling Thin Film Oven Test apparatus, model James Cox and Sons CS 325-B (Colfax, CA, USA), maintained at 163 °C for 85 min. The procedure was applied to the reference polymeric asphalt binder sample (N0) and to all nanocomposites developed with different nanomaterial contents (N2, N4, N6, N8, N10, and N12). In addition, mass loss (%) was determined for each sample, adopting a maximum acceptable limit of 1% [72]. Mass loss was calculated from the difference in sample mass before and after RTFOT aging, multiplied by 100 to express the result as a percentage. This evaluation is essential for verifying the volatilization of binder components and ensuring the thermal stability of the nanocomposites under temperature conditions representative of asphalt paving operations.

#### 3.1.3. X-Ray Diffraction

The X-ray diffraction (XRD) technique was employed to investigate the structural characteristics of the asphalt binder and to verify the incorporation of the crystalline phases of ZnO and TiO_2_ into the polymeric asphalt binder matrix. The analyses were performed using a Rigaku Miniflex II diffractometer (Akishima, Tokyo, Japan). All matrices (N0, N2, N4, N6, N8, N10, and N12) were evaluated under short-term aging conditions (RTFOT), with one sample tested for each condition. For comparison purposes, the ZnO and TiO_2_ nanomaterials were also analyzed individually. The scans were conducted over the 2θ range of 5° to 90°, with a scanning rate of 0.025°/s.

#### 3.1.4. Fourier-Transform Infrared Spectroscopy

Fourier-transform infrared spectroscopy (FTIR) was employed to investigate possible chemical interactions between the polymeric asphalt binder matrix and the incorporated nanomaterials, aiming to identify potential structural modifications or the formation of new chemical bonds resulting from the nanomodification process. The analyses were performed using a Bruker FT-IR VERTEX 70 spectrometer (Billerica, MA, USA), selected for its high sensitivity and spectral precision. Representative samples of each asphalt binder matrix (N0, N2, N4, N6, N8, N10, and N12), all previously aged through RTFOT, were analyzed. For comparison and identification of characteristic nanomaterial bands, ZnO and TiO_2_ were also tested individually. Spectra were collected from 400 to 4000 cm^−1^, covering the main vibrational bands of the polymeric asphalt binder and the nanomaterials. Each spectrum was obtained from 16 scans at a resolution of 4 cm^−1^, ensuring suitable optimization of acquisition time relative to data quality.

### 3.2. Analysis of Thermal Conductivity and Rheological Behavior to Define the Optimum Content

The analysis of thermal conductivity and high-temperature rheological behavior was conducted to assess the effects of nanomodification on the thermal and viscoelastic properties of the polymeric asphalt binder. These evaluations were fundamental for defining the optimized incorporation content of the metallic oxides (Section 3.2.3), ensuring a favorable combination between thermal enhancement and rheological compatibility under high-temperature conditions. The incorporation range was limited to a maximum of 12 wt.%, a value commonly used in heterogeneous photocatalysis formulations, allowing the combination of photocatalytic functionality with improved mechanical performance of the binder.

#### 3.2.1. Thermal Conductivity

The thermal conductivity test aimed to verify whether the heat conduction capacity of the nanomaterials was effectively transferred to the polymeric asphalt binder after nanomodification. This property is fundamental for thermal healing, as higher conductivity favors heat redistribution within the matrix, increasing the local fluidity of the binder and facilitating the diffusion of bituminous molecules in damaged zones, resulting in greater healing efficiency under elevated temperatures [69].

**Figure 5:**
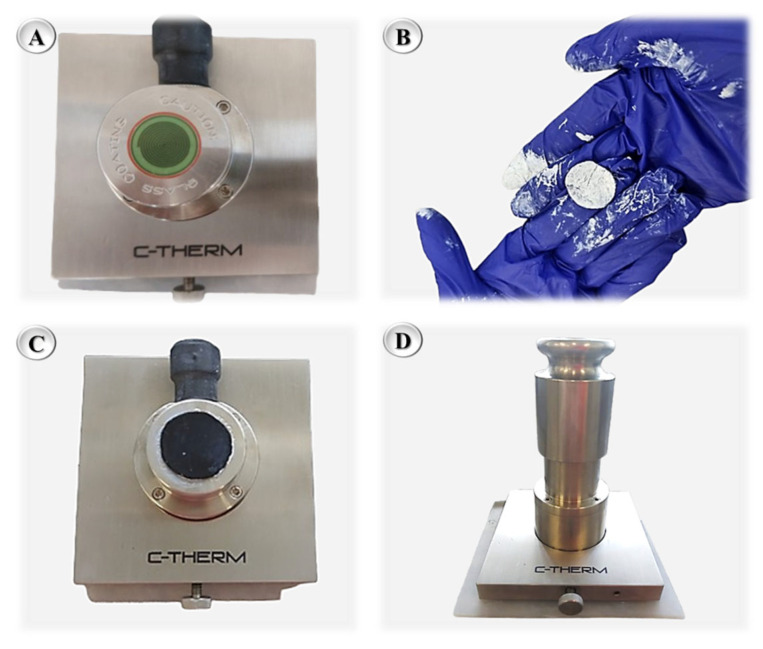
Test procedure for obtaining thermal conductivity: (**A**) highlighting the MTPS sensor of the Thermal Conductivity Analyzer equipment; (**B**) applying thermal grease to the sample to be tested; (**C**) placing the sample on top of the MTPS sensor; and, (**D**) positioning the weight on top of the sample and performing the test.

Thermal conductivity was measured using a C-Therm Thermal Conductivity Analyzer (Fredericton, NB, Canada). Cylindrical samples (25 mm diameter, 1 mm thickness) were prepared for each binder matrix (N0, N2, N4, N6, N8, N10, and N12) in the short-term aged condition (RTFOT). Six sequential measurements were performed for each sample at a controlled temperature of 25 ± 0.5 °C to ensure reproducibility. As shown in Figure 5, a thin layer of thermal grease (Thermal Joint CompoundType 120, Wakefield Thermal, Nashua, NH, USA) was applied to the sample surface to improve thermal contact with the sensor. The sample was then positioned on the sensor and loaded with a standard metallic weight to ensure proper seating and minimize measurement interferences. After preparation, sequential readings were recorded, providing a detailed profile of the thermal.

#### 3.2.2. Rheological Behavior at High Temperatures

The high-temperature rheological behavior of the asphalt matrices was evaluated using three tests: apparent viscosity, high-temperature performance grade (PGH), and multiple stress creep and recovery (MSCR). The procedures and parameters adopted for each test are described in the following subsections.

##### Apparent Viscosity

The apparent viscosity of the asphalt matrices was determined to evaluate their resistance to flow under different temperature and shear conditions, simulating real processing scenarios of the asphalt binder such as handling, mixing, and field application. This rheological parameter is essential to ensure the proper performance of the material during paving operations. The test followed ASTM D4402M [73] using spindle 21 and a Brookfield rotational viscometer, model RVDV-I+ (Middleboro, MA, USA). Seven formulations (N0, N2, N4, N6, N8, N10, and N12) were tested in the unaged condition. The apparent viscosity was measured at 135 °C, 150 °C, and 177 °C, temperatures commonly used to characterize binder flow behavior in asphalt industry. During testing, the rotational speed was adjusted to maintain torque within 10–98% of the instrument capacity, ensuring compliance with the standard validity criteria [73].

##### High-Temperature Performance Grade

The high-temperature performance grade (PGH) was determined to classify both the reference polymeric asphalt binder and the nanomodified composites with respect to their resistance to permanent deformation under elevated temperatures. The test followed ASTM D7175 [74], which specifies the procedure for evaluating the viscoelastic behavior of asphalt binders using oscillatory rheometry. A dynamic shear rheometer (DSR), model Discovery HR-2 from TA Instruments (New Castle, DE, USA), was used for the analyses. Formulations N0, N2, N4, N6, N8, N10, and N12 were evaluated in both unaged and short-term aged conditions (RTFOT). For each formulation, two specimens with a diameter of 25 mm and a 1 mm gap were tested. The PGH was determined by identifying the highest temperature at which the binder satisfies the |G*|/sin δ criterion defined in the standard. Additionally, the aging index was calculated as the ratio between |G*|/sin δ values of aged and unaged samples (Equation (1)), enabling assessment of the materials’ susceptibility to thermally induced oxidative hardening.

(1) $$\mathrm{AI}=\frac{|G*|/\sin\;\delta_{\mathrm{after-}\mathrm{RTFOT}}\;}{|G*|/\sin\;\delta_{\mathrm{before-}\mathrm{RTFOT}}\;}$$

where

AI: aging index (dimensionless);

|G*|/sin δ_after-RTFOT_: |G*|/sin δ parameter after short-term aging (kPa);

|G*|/sin δ_before-RTFOT_: |G*|/sin δ parameter before short-term aging (kPa).

##### Multiple Stress Creep and Recovery

The multiple stress creep and recovery (MSCR) test was performed to evaluate the susceptibility of asphalt binders to permanent deformation under repeated loading at high temperatures. This test allows analysis of the elastic recovery capacity and resistance to creep of the binder. The procedure followed ASTM D7405 [75], using a dynamic shear rheometer (DSR), model Discovery HR-2 from TA Instruments (New Castle, DE, USA). The reference binder (N0) and the nanocomposites N2, N4, N6, N8, N10, and N12, all previously subjected to short-term aging (RTFOT), were tested in duplicate using a 25 mm plate and a 1 mm gap. The tests were performed at 76 °C and 82 °C, temperatures selected based on the high-temperature performance grades (PGH) obtained in the previous section. These temperatures represent the upper performance limits for the base binder (PGH 76-XX) and for the nanomodified binders (PGH 82-XX), respectively.

#### 3.2.3. Definition of the Optimized Content of ZnO + TiO_2_

To investigate the contribution of nanoparticles to the healing capacity and mechanical performance of the asphalt mixture, the incorporation content of ZnO and TiO_2_ nanoparticles in equal proportions (50/50 wt.%) within the polymer-modified asphalt binder was optimized. The determination of the optimum content was based on three main criteria: (i) enhancement of thermal conductivity, since this parameter directly influences the healing phenomenon in asphaltic materials by facilitating heat diffusion and binder mobilization in damaged regions; (ii) improvement of rheological parameters at high temperatures, aiming to maintain high performance of the mixture under these critical conditions where the healing process tends to be more active due to greater molecular mobility; and (iii) limitation of the apparent viscosity to a maximum of 3.0 Pa·s at 135 °C, as established by the SUPERPAVE methodology guidelines, ensuring adequate binder workability. This approach aimed to develop a mixture that not only exhibits strong mechanical performance under elevated temperatures but also leverages these conditions to enhance the healing process. Such a strategy seeks to prevent failures such as permanent deformation precisely under conditions where healing could be most effective. After determining the optimized nanoparticle content, complementary analyses were performed for this formulation, including the evaluation of phase angle (δ) and dynamic shear modulus (|G*|) behavior at intermediate temperatures, as well as the Linear Amplitude Sweep (LAS) test to characterize the fatigue resistance of the nanocomposite.

##### Phase Angle and Dynamic Shear Modulus Behavior at Intermediate Temperatures

The rheological behavior at intermediate temperatures was evaluated to characterize the viscoelastic response of the asphalt matrices through the phase angle (δ), dynamic shear modulus (|G*|), and their respective elastic (G′) and viscous (G″) components. These parameters provide detailed information on the elastic and viscous behaviors of asphalt binders, particularly within typical service temperature ranges. The tests followed AASHTO T 315 [76] using a Discovery HR-2 hybrid dynamic shear rheometer from TA Instruments (New Castle, DE, USA). One sample of the reference binder (N0) and one sample containing the optimal nanoparticle content (N_optimal_) were tested in the short-term aged condition (RTFOT), using an 8 mm diameter and 2 mm gap geometry. The experimental protocol followed the same methodology applied in previous studies [77,78,79]. The analysis was conducted through a frequency sweep ranging from 0.1 Hz to 30 Hz to capture the material response at different loading scales. The test temperatures were set at 5 °C and 10 °C, with a strain amplitude of 0.1% (suitable for maintaining the linear viscoelastic regime under stiffer conditions), and at 15 °C, 20 °C, 25 °C, 30 °C, and 35 °C, with a strain amplitude of 1% (appropriate for temperatures where the binder exhibits lower stiffness).

##### Linear Amplitude Sweep

The linear amplitude sweep (LAS) test was conducted to evaluate the fatigue performance of the nanocomposite with the optimal nanoparticle content (N_optimal_) by analyzing the viscoelastic response of the material under progressively increasing strain levels. This test allows the estimation of the service life of the asphalt binder under cyclic loading conditions. The procedure followed AASHTO T 391 [80], applying a linear strain ramp using a Discovery HR-2 hybrid dynamic shear rheometer from TA Instruments (New Castle, DE, USA). Six samples of each material (reference binder and optimized nanocomposite), all in the short-term aged condition (RTFOT), were tested using an 8 mm diameter and 2 mm gap geometry. The test was conducted at 20 °C, adopting a 35% reduction in |G*|sin δ as the failure criterion. The experimental data were processed using the “AASHTO T 391-20—Version 1.59” [80] spreadsheet from the Modified Asphalt Research Center (MARC), University of Wisconsin–Madison (USA), to determine the rheological parameters used to construct fatigue curves (Equation (2) [80]) and to calculate the asphalt binder fatigue factor (FF) (Equation (3) [81]).

(2) $$\mathrm{Nf}=A\;\times \;({\gamma )}^{-B}$$

where

A and B: fatigue curve coefficients;

Nf: number of cycles until rupture;

γ: applied shear strain, expressed as a percentage (%).

(3) $$\mathrm{FF}=\frac{\log\;\left({Nf}_{1.25}\times {Nf}_{2.5}\right)}{2}\times \;\log\left(\frac{0.0250}{0.0125}\right)$$

where

FF: asphalt binder fatigue factor;

log: logarithm to base 10;

Nf_1.25_: number of cycles until rupture for a shear strain of 1.25%;

Nf_2.5:_ number of cycles until rupture for a shear strain of 2.5%.

### 3.3. Assessment of the Mechanical Performance of Asphalt Mixtures

The mechanical performance of the asphalt mixtures was evaluated through a series of laboratory tests, including resistance to permanent deformation, rheological behavior at intermediate temperatures (phase angle and dynamic modulus), fatigue resistance, healing capacity, and the assessment of internal heating rate under microwave radiation. The reference asphalt mixture produced with the reference polymeric binder (N0) is denoted as M1, while the nanomodified asphalt mixture, produced using the optimized ZnO + TiO_2_ nanocomposite (N_optimal_) determined in Section 3.2.3, is designated as M2.

The binder content adopted for both mixtures was the same as that established by Manfro et al. [36], since the constituent materials, including mineral aggregates, gradation curve, and the reference asphalt binder, were identical to those used in the present study. This ensured uniform production conditions, eliminating the influence of binder content on the comparative analysis of the mechanical performance of the formulations. Both mixtures were produced with 4.44% asphalt binder. Sample compaction was performed using a SUPERPAVE gyratory compactor, model Servopac, manufactured by IPC Global^®^ (Boronia, Victoria, Australia), currently part of the Controls Group. The compaction pressure was maintained at 600 ± 18 kPa, with an external angle of 1.25 ± 0.02° and a rotation speed of 30 ± 0.5 rpm. The compaction protocol followed the parameters defined for high traffic volume (≥30 million ESALs), adopting the following criteria: N_initial_ = 9, N_design_ = 125, and N_max_ = 205. The volumetric properties of the compacted mixtures met the requirements specified for a nominal maximum aggregate size of 19 mm, as defined for mixtures intended for extremely high traffic (prior to SUPERPAVE 5). The gradation used in the formulations is presented in Table 6 [82]. All mixture design and preparation procedures strictly followed the guidelines established in AASHTO M 323 [83], AASHTO R 35 [84], and AASHTO R 30 [85].

**Figure 6:**
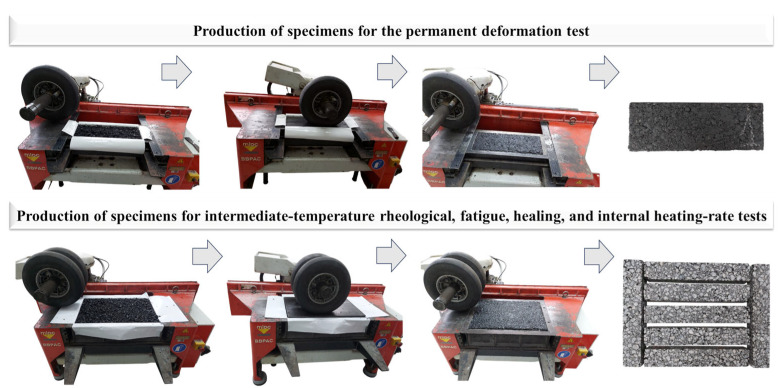
Production process of asphalt mixture specimens using the French wheel compactor. The (**upper**) sequence illustrates the procedure for producing specimens for the permanent deformation test, while the (**lower**) sequence represents the production of slabs for intermediate-temperature rheological, fatigue, healing, and internal heating-rate tests.

In the subsequent stage, the asphalt mixtures were compacted using the French BBPAC compaction table (MLPC^®^), developed by the company VECTRA (currently NextRoad), based in Fontaine-lès-Dijon, Bourgogne-Franche-Comté, France. The compaction process followed the specifications outlined in EN 12697—Part 33 [86]. After compaction, the specimens intended for the rutting test measured 50 cm × 18 cm × 5 cm. The slabs designated for intermediate-temperature rheological, fatigue, healing, and internal heating-rate tests were initially molded with dimensions of 60 cm × 40 cm × 9 cm. These slabs were later cut into specimens with final dimensions of 38.1 cm × 6.35 cm × 5.08 cm, in accordance with the geometries required for the respective mechanical tests. The compaction, preparation, and cutting procedures of the test specimens are schematically illustrated in Figure 6 to visually represent the experimental stages involved.

#### 3.3.1. Permanent Deformation Resistance

The rutting test was conducted to determine the resistance of the nanomodified asphalt mixture (M2) to wheel-tracking deformation. The performance related to this property was evaluated according to the procedure established in EN 12697—Part 22 [87], using the Orniéreur (MLPC^®^) equipment developed by the company VECTRA (currently NextRoad), based in Fontaine-lès-Dijon, Bourgogne-Franche-Comté, France. For each asphalt mixture, two specimens were tested under a controlled temperature of 60 °C, with a longitudinal load of 5000 ± 50 N applied at a frequency of 1 Hz. Subsequently, the rut depth percentage of mixtures M1 and M2 was calculated according to Equation (4) for the following load cycles: 100, 300, 1000, 3000, 10,000, and 30,000.

(4) $$\mathrm{Pi}=100\;\times \frac{\Sigma j\left(\mathrm{mij}-m0j\right)}{15\;\times \;\mathrm{ES}}$$

where

**Figure 7:**
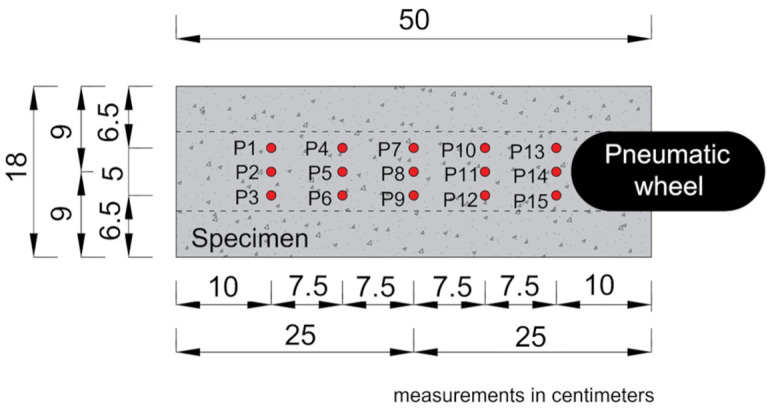
Representation of the allocation of points P1 to P15 for measuring the depth of the specimen rutting (adapted of Melo [82]).

P_i_: mean percentage of sinking on the plate surface in cycle i (%);

j: permanent deformation reading point (points P1 to P15 according to Figure 7);

m_ij_: depth measurement in cycle i, point j;

m_0j_: depth measurement in cycle 0, point j;

ES: plate height.

#### 3.3.2. Rheological Behavior at Intermediate Temperatures

The rheological behavior of the asphalt mixtures at intermediate temperatures was evaluated through the analysis of the phase angle (δ) and the dynamic modulus (|E*|), which are key parameters for characterizing the viscoelastic response of the material under cyclic loading. The experimental procedure followed the requirements established in EN 12697—Part 26 [88], which specifies the determination of the viscoelastic properties of asphalt mixtures using four-point bending beam tests. The tests were carried out using the Pneumatic 4 Point Bending Apparatus manufactured by IPC Global^®^ (Boronia, Victoria, Australia), currently part of the Controls Group. Two specimens were tested: one corresponding to the reference asphalt mixture (M1) and the other to the nanomodified mixture (M2). The test temperatures adopted were 0 °C, 5 °C, 10 °C, 15 °C, 20 °C, 25 °C, and 30 °C, covering a representative range of field operating conditions. For each temperature, different loading frequencies were applied (0.1 Hz, 0.2 Hz, 0.5 Hz, 1 Hz, 2 Hz, 5 Hz, 10 Hz, 15 Hz, and 20 Hz) to generate a broad response spectrum of the viscoelastic behavior of the mixtures [88,89].

#### 3.3.3. Fatigue Resistance and Healing Capacity

The evaluation of fatigue resistance and healing capacity of the asphalt mixtures was performed in three sequential stages: (1) execution of the first fatigue test; (2) application of the healing protocol, according to the methodology described by [37]; and (3) execution of a second fatigue test to assess the residual life of the asphalt mixture after the healing process. The fatigue tests were conducted in accordance with the procedures established in EN 12697—Part 24 [90], using the Pneumatic 4 Point Bending Apparatus by IPC Global^®^ (Boronia, Victoria, Australia). A total of 43 specimens were tested, each with dimensions of 38.1 cm × 6.35 cm × 5.08 cm, including 20 specimens from the reference asphalt mixture (M1) and 23 specimens from the nanomodified mixture (M2). The test parameters adopted were: temperature of 20 °C, sinusoidal loading frequency of 10 Hz, controlled microstrain levels between 225 µm/m and 293 µm/m, and the failure criterion defined by the standard as a 50% reduction in the initial dynamic modulus, with this value measured at the 100th load cycle [90]. The characteristic fatigue curve model for each mixture was fitted using Equation (5), while the fatigue factor of the asphalt mixtures (FFM) was calculated using Equation (6), as proposed by [91].

(5) $$\mathrm{Nf}=a\;\times \;\varepsilon^{b}$$

where

Nf: number of load applications until the initial dynamic modulus decreases by 50%;

ε: specific tensile strain (microstrain);

“a” and “b”: constants.

(6) $$\mathrm{FFM}=0.2\times \left[\log({\mathrm{Nf}}_{100})+\log({\mathrm{Nf}}_{250})\right]$$

where

FFM: fatigue factor of the asphalt mixture;

log: base-10 logarithm;

Nf_100_: number of load applications for the specific microstrain of 100 µm/m;

Nf_250_: number of load applications for the specific microstrain of 250 µm/m.

After completing the first fatigue test, the same specimens were subjected to a healing protocol developed by Schuster [37], with the aim of investigating the healing capacity of the asphalt mixtures. The protocol consisted of the following stages: (1) microwave heating for 140 s immediately after the completion of the first fatigue test, promoting partial mobilization of the binder constituents and activation of physicochemical self-healing mechanisms; (2) thermal conditioning in the climatic chamber of the fatigue testing equipment after heating, where the specimens were kept at rest for 3 h under a controlled temperature of 20 °C; (3) after 2 h and 45 min of rest, repositioning of the specimen in the fatigue testing apparatus; and (4) after the 3 h rest period, resumption of the fatigue test (second test) using the same parameters as the initial test (temperature, frequency, microstrain, and loading mode) until the failure criterion was reached, that is, a 50% reduction in the initial dynamic modulus, measured at the 100th cycle of the first fatigue test. Finally, based on the obtained results, Equations (7) and (8) were used to calculate the healing percentage and the normalized healing, respectively.

(7) $$\%H=\;\frac{{\mathrm{Nf}\;}_{2\mathrm{nd}\;\mathrm{fatigue}\;\mathrm{test}}}{{\mathrm{Nf}\;}_{1\mathrm{st}\;\mathrm{fatigue}\;\mathrm{test}}}\times 100\;$$

where

%H: healing percentage;

Nf 1st _fatigue test_: number of cycles until reaching 50% of the initial dynamic modulus;

Nf 2nd _fatigue test_: number of cycles after the healing procedure, until the stopping criterion is reached.

(8) $$\mathrm{NH}=\frac{\%H}{ED}$$

where

NH: normalized healing [1/(J/m^3^)];

%H: healing percentage;

ED: energy density (J/m^3^).

#### 3.3.4. Assessment of the Internal Heating Rate of Asphalt Mixtures Under Microwave Radiation

This stage aimed primarily to evaluate the internal heating rate of the reference asphalt mixture (M1) and the nanomodified mixture (M2) when subjected to microwave heating. The objective was to investigate whether the incorporation of metallic oxides contributed to increasing the thermal efficiency of the asphalt matrix, thereby enhancing its response to heat application.

**Figure 8:**
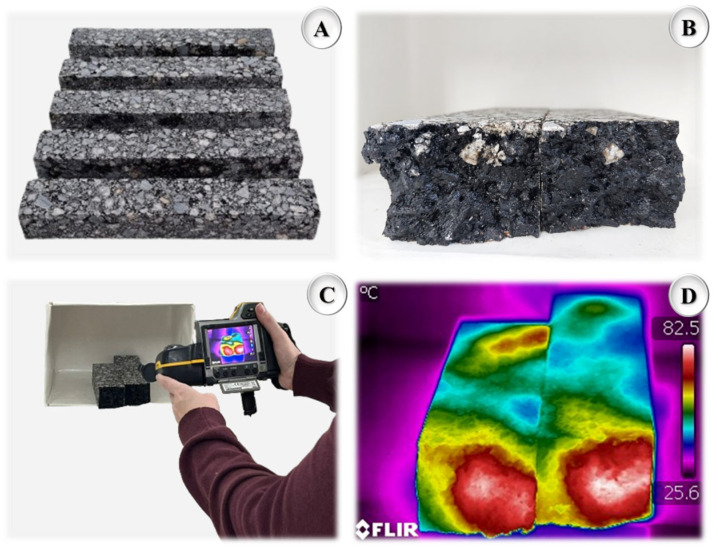
Heating and temperature measurement procedure (**A**) test specimens with dimensions of 38.1 cm × 6.35 cm × 5.08 cm before the heating and breaking process (**B**) bipartite test specimen for measuring the internal temperature (**C**) thermographic recording with the FLIR^®^ brand camera (Teledyne FLIR LLC, Wilsonville, OR, USA) and (**D**) thermographic image of the bipartite test specimen.

For this purpose, specimens (38.1 cm × 6.35 cm × 5.08 cm) of each mixture were preconditioned for at least 24 h at a constant temperature of 20 °C to ensure uniform initial thermal conditions. The specimens were then heated in a microwave oven (Electrolux, model ME28S, nominal power of 900 W, frequency of 2450 MHz, and internal volume of 28 L) (Manaus, Amazonas, Brazil) for six different exposure times: 30 s, 60 s, 90 s, 120 s, 150 s, and 183 s (three specimens for each heating duration). Immediately after heating, each specimen was longitudinally sectioned, and the internal temperature was measured using an infrared thermographic camera (FLIR^®^ model B400, Wilsonville, OR, USA). From the collected data, mean internal temperature curves (Equation (9)) were plotted as a function of heating time, allowing for a comparative evaluation of the thermal performance of both formulations. Based on the results, it was possible to determine whether the presence of metallic oxides, due to their electromagnetic properties and thermal conductivity, enhanced the absorption and conversion of microwave energy into heat, promoting faster and deeper heating within the asphalt matrix. This characteristic is particularly relevant, as it helps explain potential improvements in healing capacity discussed in the previous section. A higher internal heating rate facilitates the mobilization of the asphalt binder and accelerates the processes of flow and recombination of molecular bonds, key factors for the healing of microcracks and the partial restoration of the functional properties of the asphalt matrix. Figure 8 illustrates the heating, sectioning, and thermal imaging stages.

(9) $$\mathrm{MIT}=\;\frac{\;H+L}{2}$$

where

MIT: mean internal temperature (°C);

H: highest test specimen temperature (°C);

L: lowest test specimen temperature (°C).

## 4. Results and Discussion

This section presents and discusses the experimental results, beginning with the nanomodification of the polymeric asphalt binder, followed by the analysis of thermal conductivity and rheological behavior to determine the optimum nanoparticle content, and concluding with the evaluation of the mechanical performance of the reference and nanomodified asphalt mixtures.

### 4.1. Nanomodification of the Polymeric Asphalt Binder

The nanomodification process of the base polymer-modified asphalt binder (N0) resulted in the formulation of six nanocomposites with different incorporation levels of ZnO and TiO_2_, in a 50/50 wt.% ratio, designated as N2, N4, N6, N8, N10, and N12. After preparation, the nanocomposites were first examined using bright-field microscopy to assess the dispersion of the nanomaterials within the asphalt matrix. Subsequently, short-term aging (RTFOT) was performed to simulate the initial degradation conditions that occur during asphalt mixture production [71]. The nanocomposites were then characterized with respect to their internal structure, including the evaluation of structural and crystalline features by X-ray diffraction (XRD) and the identification of functional groups using Fourier-transform infrared spectroscopy (FTIR). These techniques provided a comprehensive characterization of the materials, enabling a deeper understanding of the nanomaterial incorporation process into the polymeric asphalt binder and offering essential information for interpreting the mechanical and rheological behavior of the matrices, discussed in the subsequent sections of this study.

#### 4.1.1. Bright-Field Microscopy

**Figure 9:**
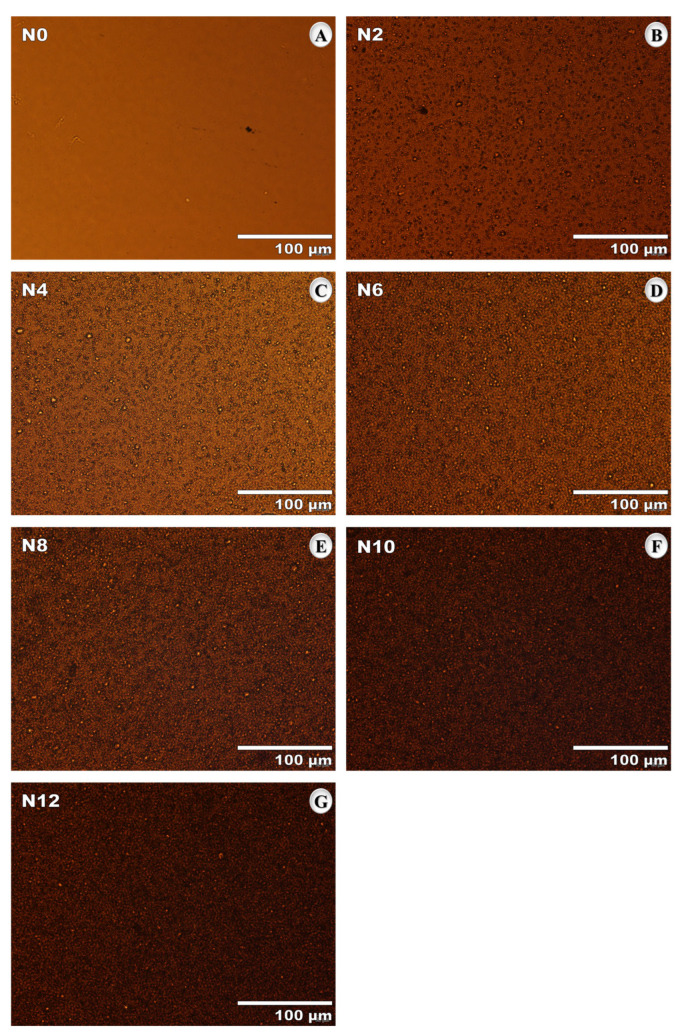
Bright-field microscopy images of samples in the unaged state: (**A**) reference asphalt binder N0 (0% ZnO + TiO_2_) and nanocomposites with varying concentrations of ZnO + TiO_2_: (**B**) N2 (2% ZnO + TiO_2_), (**C**) N4 (4% ZnO + TiO_2_), (**D**) N6 (6% ZnO + TiO_2_), (**E**) N8 (8% ZnO + TiO_2_), (**F**) N10 (10% ZnO + TiO_2_), and (**G**) N12 (12% ZnO + TiO_2_).

The micrographs shown in Figure 9A–G indicate a uniform distribution of the nanomaterials within the matrix, even at higher concentrations, with no evidence of significant agglomerate formation. This behavior is particularly relevant, as proper dispersion of the nanomaterials can directly contribute to improved structural uniformity of the matrix and, consequently, to enhanced mechanical performance.

#### 4.1.2. Short-Term Aging

**Figure 10:**
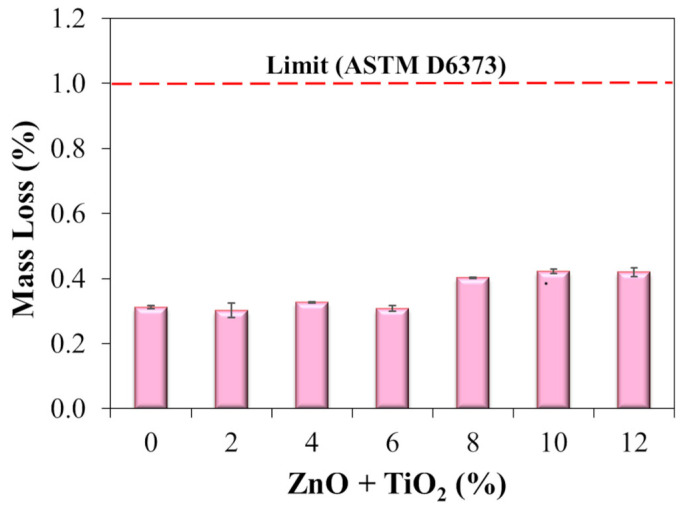
Mass loss of reference asphalt binder and nanocomposites produced after short-term aging simulation performed with Rolling Thin-Film Oven Test (RTFOT) [71,72]. Error bar: standard deviation.

The mass loss after short-term aging (RTFOT) is presented in Figure 10. It can be observed that all nanocomposites, regardless of the ZnO + TiO_2_ incorporation level, exhibited mass loss values below 1%, thus meeting the maximum limit established by ASTM D6373 [72]. This result indicates that nanoparticle modification did not compromise the stability of the asphalt binders during the initial aging process. The observed mass loss is associated with the volatilization of light compounds present in the asphalt binder, which tend to evaporate under the thermal conditions of the RTFOT [71]. However, variations among the different nanocomposites were minor and did not show any systematic trend related to the ZnO + TiO_2_ content. Such variations may be attributed to the inherent uncertainty of the testing method, which involves processes such as evaporation and air flow, both susceptible to slight operational fluctuations. Maintaining the mass loss within acceptable limits in all cases suggests that the presence of ZnO and TiO_2_ did not intensify volatile emissions.

#### 4.1.3. X-Ray Diffraction

**Figure 11:**
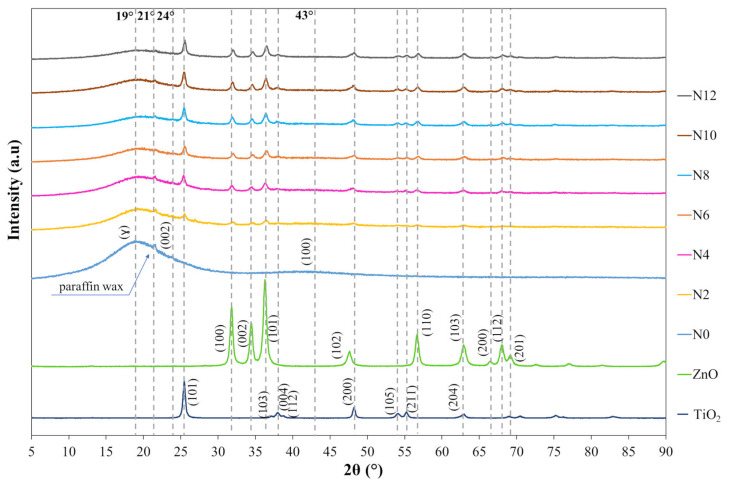
X-ray diffraction patterns of the nanomaterials (ZnO and TiO_2_) and of the samples in the short-term aging state of the reference asphalt binder N0 (0% ZnO + TiO_2_) and of the nanocomposites with varying concentrations of ZnO + TiO_2_: N2 (2%), N4 (4%), N6 (6%), N8 (8%), N10 (10%) and N12 (12%). Dashed vertical lines: indicate the 2θ positions corresponding to the main diffraction peaks of ZnO, TiO_2_, and the reference asphalt binder (N0).

Figure 11 presents the X-ray diffraction (XRD) results obtained for the nanomaterials (ZnO and TiO_2_), the reference asphalt binder (N0), and the nanocomposites N2, N4, N6, N8, N10, and N12, all under short-term aging conditions (RTFOT). The obtained data allow for the characterization of the crystalline structure of the materials and the examination of the interaction between the oxides and the asphalt matrix after the incorporation process.

In Figure 11 the diffraction patterns of the TiO_2_ nanoparticles predominantly indicate the anatase phase (tetragonal structure), consistent with the JCPDS (Joint Committee on Powder Diffraction Standards) card 21-1272 [6,20,92,93]. For ZnO, the wurtzite phase (hexagonal lattice) is observed, in accordance with JCPDS 79-2205 [6,20,94,95]. The absence of additional reflections suggests high purity of the nanomaterials, with no evidence of impurities or secondary phases.

Regarding the reference polymeric asphalt binder (N0), the diffractogram exhibits a broad amorphous band at 2θ = 19° (γ band), associated with the presence of stacked aliphatic chains in the asphaltene structure [96,97], and a band at 2θ = 24° (002), related to the stacking of aromatic rings, also characteristic of asphalt binders [97]. A distinct peak at 2θ = 21° is also observed, attributable to paraffin wax [98], along with a band around 2θ = 43° (100), associated with the size of sheets formed by fused aromatic rings [96].

With the incorporation of ZnO and TiO_2_ nanomaterials into the polymeric asphalt binder (N0), a systematic reduction in the relative intensity of the characteristic asphalt binder bands (γ, 002, and 100) is observed as the incorporation level increases. This reduction is consistent with the dilution and attenuation effects of the diffracted signal of the binder caused by the presence of the added inorganic crystalline phases, without measurable changes in the positions (2θ) or full width at half maximum (FWHM) of the main bands. Thus, there is no evidence of detectable structural modifications in the mean stacking of the aromatic and aliphatic constituents of the binder.

In parallel, crystalline peaks attributed to the incorporated oxide phases emerge and become more pronounced. For ZnO, the most prominent regions correspond to hkl (100) and (101), consistent with the isolated material; for TiO_2_, the highest intensity occurs at (101), typical of the anatase crystalline phase, which is also dominant in the pure oxide sample. The clear and increasing presence of these crystalline phases in the nanocomposites, without significant overlapping or shifting of diffraction peaks, suggests a good physical distribution of the nanomaterials within the asphalt matrix, with no evidence of chemical reactions between the constituents. This indicates that the incorporation process preserves the structural integrity of the components, characterizing a predominantly physical interaction consistent with the objective of nanometric reinforcement. Such structural compatibility is essential to ensure the stability and functionality of the nanocomposites.

Finally, the observed reduction in the amorphous band (associated with the reference asphalt binder) may be correlated with improved dispersion of the SBS polymer. This behavior supports the hypothesis that the nanomodification process not only promotes the incorporation of nanomaterials but also contributes to a more uniform internal microstructure.

#### 4.1.4. Fourier-Transform Infrared Spectroscopy

**Figure 12:**
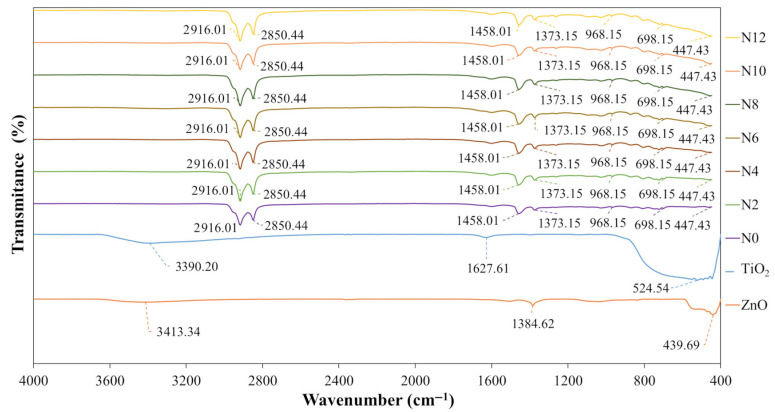
FTIR spectra of the nanomaterials (ZnO and TiO_2_) and of the samples in the short-term aging state of the reference asphalt binder N0 (0% ZnO + TiO_2_) and of the nanocomposites with varying concentrations of ZnO + TiO_2_: N2 (2%), N4 (4%), N6 (6%), N8 (8%), N10 (10%) and N12 (12%).

Figure 12 presents the Fourier-transform infrared (FTIR) spectra obtained for the nanomaterials (ZnO and TiO_2_), the reference asphalt binder (N0), and the developed nanocomposites (N2, N4, N6, N8, N10, and N12), all analyzed under short-term aging conditions (RTFOT). These results enable the comparison of the spectral signatures of the oxides and the polymeric matrix, as well as the evaluation of the effects of nanomaterial incorporation on the chemical structure of the asphalt binder.

To contextualize the isolated nanomaterials, Figure 12 shows that ZnO exhibits a peak at 439.68 cm^−1^, attributed to the stretching vibration of the Zn–O bond, characteristic of the wurtzite structure [99,100,101], as well as a peak at 1384.62 cm^−1^ associated with the symmetric stretching vibration mode of the carboxylate group (COO^−^), possibly related to residual organic traces from synthesis or stabilization [102]. A broad band is also observed at 3413.34 cm^−1^ (O–H stretching), typical of hydroxyl groups or adsorbed water molecules on the surface of high–surface-area materials [99,101,102,103]. In turn, TiO_2_ exhibits a peak at 524.54 cm^−1^, corresponding to the Ti–O–Ti bridge stretching vibrations, consistent with the anatase/rutile phases [104,105,106], as well as a band at 1627.61 cm^−1^, associated with angular deformation (bending) and stretching vibrations of hydroxyl groups (–OH), commonly attributed to the presence of chemisorbed water on the surface of materials with high specific surface area [105]. In addition, the band observed at 3390.20 cm^−1^ corresponds to symmetric and asymmetric stretching vibrations of the –OH group, indicating the presence of hydroxyl groups strongly bonded to the TiO_2_ surface [104,106].

For the reference polymeric asphalt binder (N0) (Figure 12), the peaks located at 2916.01 cm^−1^ and 2850.44 cm^−1^ are attributed in the literature to the C–H stretching vibrations of aliphatic chains [107,108]. Furthermore, the peaks at 1458.01 cm^−1^ and 1373.15 cm^−1^ correspond to vibrations associated with the –CH_2_– and –CH_3_– groups, respectively [107,108,109,110]. The lower–intensity peaks observed at 968.15 cm^−1^ and 698.15 cm^−1^ are attributed to the butadiene chain in the SBS copolymer [111] and to C–H vibrations in monoalkylated aromatics, characteristic of polystyrene [109], confirming the presence of the copolymer in the matrix.

In the modified nanocomposites (Figure 12), a gradual reduction in transmittance is observed near 447.43 cm^−1^, proportional to the increase in incorporated ZnO + TiO_2_ content. This behavior is related to the characteristic bands of the nanomaterials at 439.69 cm^−1^ for ZnO and 524.54 cm^−1^ for TiO_2_, and indicates the presence of these metallic oxides in the matrix. It is important to note that the incorporation of ZnO and TiO_2_ did not cause significant changes in the main peaks of the polymeric asphalt binder matrix, indicating that no relevant chemical reactions occurred between the components. This chemical stability suggests a predominantly physical interaction. Similar results were reported by Neto et al. [112] and Filho et al. [108] when evaluating asphalt composites modified with metallic oxides.

Finally, the preservation of the characteristic peaks of the SBS copolymer, even after the addition of nanomaterials, reinforces the hypothesis that the nanomodification process does not compromise the integrity of the polymeric modifier. The XRD and FTIR results confirm that the incorporation of ZnO and TiO_2_ preserved the structural and chemical integrity of the system, which is a fundamental condition to ensure thermal and rheological consistency in the subsequent evaluation stages. Therefore, the analysis now turns to the functional performance of the nanomodified binders, focusing on properties directly associated with service behavior, such as thermal conductivity and rheological performance.

### 4.2. Analysis of Thermal Conductivity and Rheological Behavior to Define the Optimum Content

This section presents and discusses the results related to the thermal conductivity and high-temperature rheological behavior of the developed asphalt nanocomposites. The combined analysis of these parameters allowed the evaluation of the impact of nanomodification with metallic oxides on the performance of the asphalt binder, providing technical support for defining the optimized ZnO and TiO_2_ content to be used in a dense asphalt mixture.

#### 4.2.1. Thermal Conductivity

**Figure 13:**
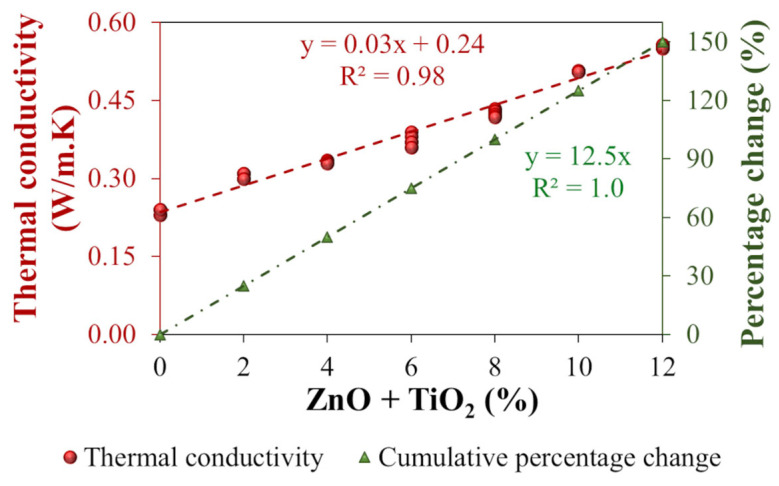
Thermal conductivity of polymeric asphalt binder and nanocomposites under short-term aging condition (RTFOT).

The thermal conductivity of the nanocomposites was evaluated to verify the effective transfer of the thermal properties of the nanomaterials (ZnO and TiO_2_) to the polymeric asphalt binder. This parameter is highly relevant, as materials with higher thermal conductivity promote the healing process of the asphalt structure, making the localized heating required to induce binder flow and close microcracks more efficient. The improvement of thermal properties through the incorporation of various types of nanomaterials into asphalt binders has been reported in the literature [69,113]. The results obtained for the nanocomposites developed in this study are presented in Figure 13.

Figure 13 shows that as the nanomaterial content in the polymeric asphalt binder increases, the thermal conductivity also rises. For the highest incorporation level of ZnO + TiO_2_ analyzed (12%), the thermal conductivity reached 0.6 W/m·K, representing a 150% increase compared with the reference polymeric asphalt binder (0%).

A one-way Welch ANOVA analysis yielded *p* < 0.001, indicating a statistically significant difference among the ZnO + TiO_2_ incorporation levels with respect to thermal conductivity. The Games–Howell post hoc test (Table 7) reinforces this evidence, with most comparisons showing *p* < 0.001 (statistically significant differences), except for the comparison between N4 and N6, which yielded *p* = 0.003 (still within the threshold for statistical significance). Furthermore, the trend observed in the mean difference values suggests that increasing the concentration of these nanomaterials contributes to enhancing the thermal conductivity of the polymeric asphalt binder.

Finally, this improvement in thermal conductivity is relevant because it promotes more efficient heat redistribution within the asphalt matrix at elevated temperatures. This behavior may facilitate binder molecule mobility and favor the closure of microcracks, a mechanism associated with the healing potential of asphalt materials.

#### 4.2.2. Rheological Behavior at High Temperatures

The following section presents and discusses the results of the rheological behavior at high temperatures, considering apparent viscosity, performance grade (PGH), and multiple stress creep and recovery (MSCR) tests.

##### Apparent Viscosity

**Figure 14:**
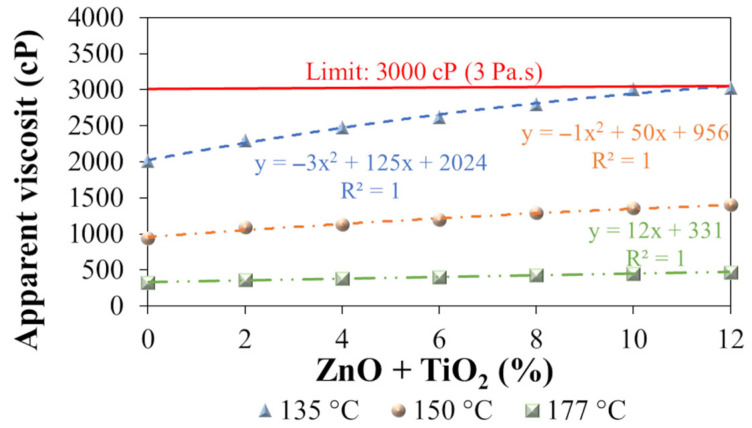
Apparent viscosity of polymeric asphalt binder and nanocomposites at temperatures of 135 °C, 150 °C and 177 °C.

The apparent viscosity was determined for both the polymeric asphalt binder (N0) and the nanocomposites (N2, N4, N6, N8, N10, and N12) at temperatures of 135 °C, 150 °C, and 177 °C (Figure 14).

At all test temperatures, a progressive increase in apparent viscosity was observed with the addition of ZnO + TiO_2_ nanoparticles to the polymeric asphalt binder. The analysis of the trend curves (Figure 14) shows that the highest percentage increase in apparent viscosity between the N0 composite (without nanomaterials) and N12 (with 12% ZnO + TiO_2_) occurs at 135 °C, with a 52.8% increase. At 150 °C and 177 °C, the observed increases were 47.7% and 43.5%, respectively. These results indicate that the effect of the nanomaterials on viscosity is more pronounced at lower temperatures, which may significantly impact the workability of the binder during production and application of the asphalt mixture.

The trend lines (Figure 14) show that at 135 °C, starting from a ZnO + TiO_2_ concentration of approximately 10.4%, the viscosity exceeds the 3 Pa·s limit established by the SUPERPAVE methodology [114] to ensure adequate pumpability and handling of the binder. Binders with viscosity values above this threshold may present operational challenges and require higher energy for pumping.

Therefore, the results demonstrate that the incorporation of ZnO + TiO_2_ nanomaterials directly affects the apparent viscosity of the polymeric asphalt binder. This behavior is consistent with previous studies that investigated the individual addition of ZnO and TiO_2_ to different asphalt matrices [14,15,62,70], which also reported viscosity increases as a result of nanometric modification.

From a functional performance perspective, this increase in viscosity can be beneficial under high-temperature service conditions, contributing to greater resistance to permanent deformation, as demonstrated by Neto et al. [14] and Cadorin et al. [62]. However, it is essential to define the nanomaterial dosage in a manner that ensures improved mechanical performance while preserving appropriate processing and application conditions.

##### High-Temperature Performance Grade

**Figure 15:**
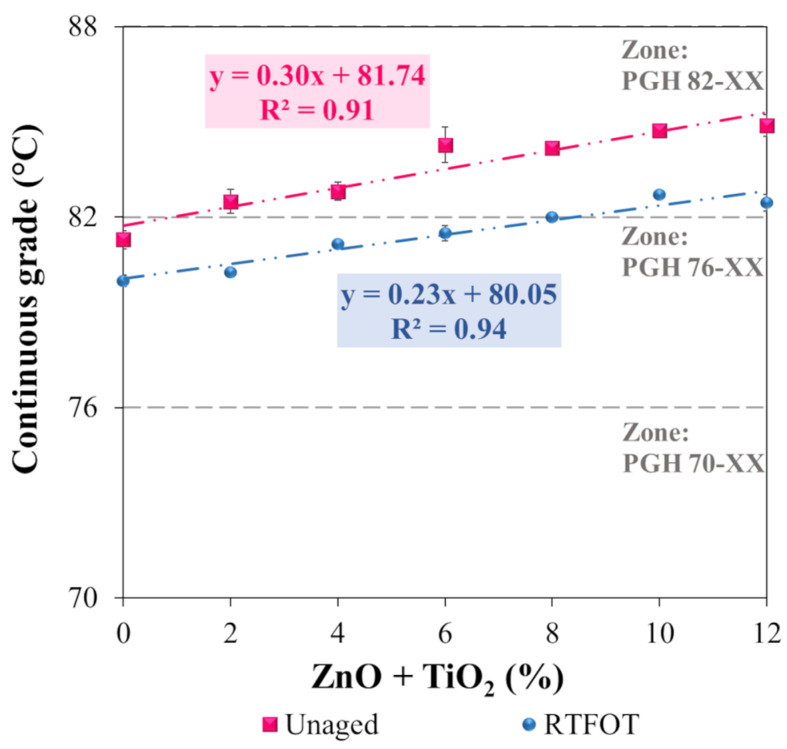
Influence of the incorporation of ZnO + TiO_2_ in the polymeric asphalt binder on the PGH classification at high temperatures, in the short-term aging (RTFOT) and unaged condition. Error bar: standard deviation.

Figure 15 shows the influence of ZnO + TiO_2_ incorporation into the polymeric asphalt binder on the high-temperature performance grade (PGH), evaluated in both the unaged and short-term aged (RTFOT) conditions.

The incorporation of ZnO + TiO_2_ nanoparticles into the polymeric asphalt binder promoted a significant increase in high-temperature resistance, expressed by the rise in continuous grade. The observed increment rates were 0.30 °C/(%ZnO + TiO_2_) for the unaged binder and 0.23 °C/(%ZnO + TiO_2_) after short-term aging (RTFOT), as evidenced by the trend lines in Figure 15. This modification directly resulted in an improvement in the performance classification of the binder.

In the unaged condition (Figure 15), the transition of the high-temperature performance grade (PGH) from 76-XX to 82-XX occurred at a minimum concentration of 0.9% ZnO + TiO_2_. After RTFOT aging, this change was observed from 8.5% nanoparticle incorporation. These results indicate that the addition of nanomaterials contributes to increasing the critical failure temperature of the polymeric asphalt binder, enhancing its performance against permanent deformation under hot climates and/or heavy traffic loads. Similar findings were reported by Neto et al. [14] for ZnO and by Babagoli et al. [7] and Cadorin et al. [62] for TiO_2_, corroborating the effectiveness of these nanomaterials in improving the thermal performance of asphalt binders.

**Figure 16:**
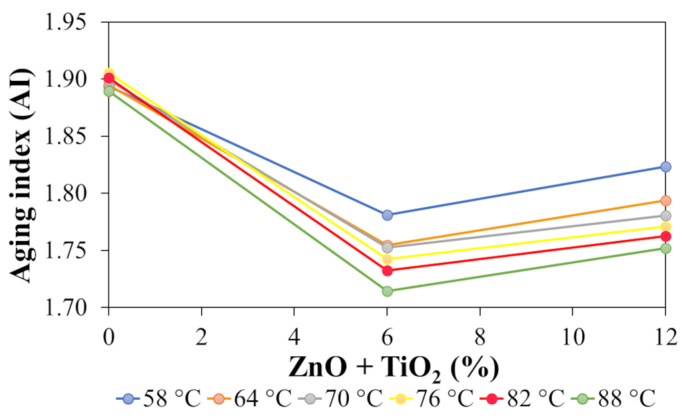
Corresponding aging index (AI) for each analyzed test temperature and incorporation content of ZnO + TiO_2_.

Based on the PGH values obtained, the aging index (AI) was also calculated to evaluate the susceptibility of the binder to aging. The index was determined specifically for samples N0 (reference), N6, and N12, considering the sensitivity of the test. Figure 16 presents the evolution of the AI as a function of temperature and ZnO + TiO_2_ concentration.

As shown in Figure 16, the progressive incorporation of ZnO and TiO_2_ nanoparticles into the polymeric asphalt binder promotes a systematic reduction in the aging index (AI), indicating lower susceptibility to short-term oxidative aging. This trend suggests that the nanomaterials act as stabilizing agents, enhancing binder durability by mitigating the detrimental effects of thermal aging.

The decrease in AI reflects a smaller variation in the critical performance temperature before and after aging (RTFOT), demonstrating an asphalt matrix less sensitive to thermo-oxidative transformations. This behavior was also observed by Neto et al. [14], who attributed the presence of ZnO to an antioxidant effect, reducing the formation of oxidative species and thereby limiting the degradation of binder constituents. Complementarily, Cadorin et al. [62] highlighted the role of TiO_2_ as a nanofiller that decreases the porosity of the asphalt matrix, increasing its impermeability and limiting both the volatilization of light components and the diffusion of oxygen, factors directly associated with the aging process. It is important to note that the reduction in AI was more pronounced up to approximately 6% ZnO + TiO_2_ incorporation, after which a stabilization and/or slight increase trend was observed.

##### Multiple Stress Creep and Recovery

**Figure 17:**
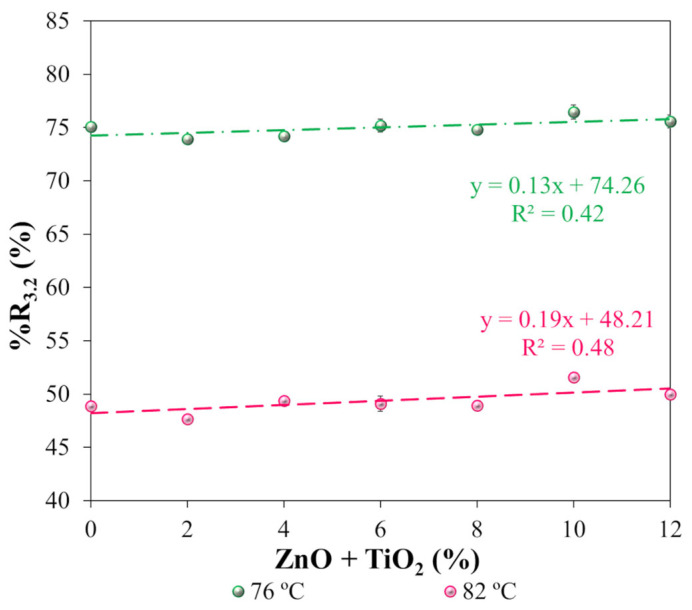
Behavior of recovery percentage (%R_3.2_) in relation to the content of ZnO and TiO_2_ incorporated in the polymeric asphalt binder. Error bar: standard deviation.

**Figure 18:**
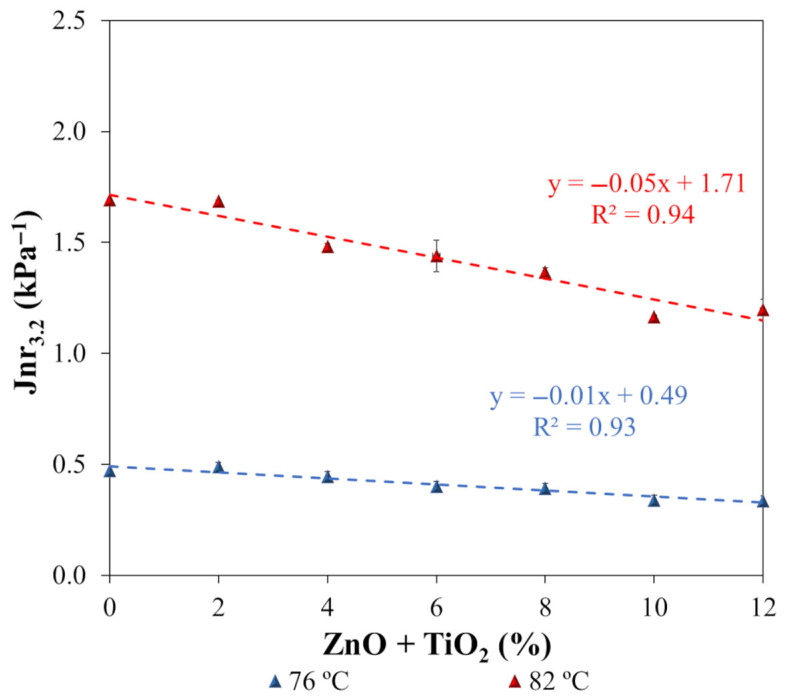
Behavior of non-recoverable compliance (Jnr_3.2_) in relation to the content of ZnO and TiO_2_ incorporated in the polymeric asphalt binder. Error bar: standard deviation.

The susceptibility of the asphalt matrices to permanent deformation was evaluated using rheological parameters obtained from the multiple stress creep and recovery (MSCR) test, with emphasis on the percentage of elastic recovery (%R_3.2_) and the non-recoverable creep compliance (Jnr_3.2_). These parameters were determined under a stress level of 3.2 kPa, applied at temperatures of 76 °C and 82 °C on samples aged using the RTFOT procedure. Figure 17 presents the %R_3.2_ values, while Figure 18 shows the corresponding Jnr_3.2_ results.

The percentage of elastic recovery (%R_3.2_), presented in Figure 17, showed absolute increases of 1.6 and 2.3 percentage points at 76 °C and 82 °C, respectively, when comparing the reference polymeric asphalt binder (N0) with the nanocomposite containing 12% ZnO + TiO_2_ (N12). Despite the observed increases, these values can be considered modest and do not exhibit a clearly defined trend as a function of the nanomaterial content. These results contrast with those reported by Günay and Ahmedzade [115], who observed an absolute increase of 1.7 percentage points in %R_3.2_ at 76 °C after incorporating 4% TiO_2_ at the nanoscale into an asphalt binder modified with 3% SBS. Similarly, Melo et al. [20] reported that the isolated addition of TiO_2_ and ZnO did not produce significant changes in the elastic response of a conventional asphalt binder (PGH 58-XX) under elevated temperatures.

Regarding the non-recoverable creep compliance (Jnr_3.2_), presented in Figure 18, a progressive reduction trend was observed with increasing ZnO + TiO_2_ nanoparticle content in the polymeric asphalt binder. This trend is evidenced by the regression curves, whose high coefficients of determination (R^2^) indicate a strong correlation between the increase in nanomaterial concentration and the reduction in binder deformability. Based on the trend line analysis, the addition of 12% ZnO + TiO_2_ (N12) resulted in decreases of 24.5% and 35.1% in Jnr_3.2_ at 76 °C and 82 °C, respectively, compared to the reference binder (N0). These results indicate a significant and systematic improvement in resistance to permanent deformation through the combined use of metallic oxide nanoparticles. Similar but isolated behaviors have been reported in the literature. Neto et al. [14] found that the addition of 7% ZnO to a conventional asphalt binder resulted in an 89.5% reduction in Jnr_3.2_ at 64 °C. Likewise, Günay and Ahmedzade [115] observed that adding 4% TiO_2_ to an SBS-modified binder reduced Jnr_3.2_ by 8.6% at 76 °C. The observed reduction in non-recoverable compliance can be attributed to the nanoscale reinforcement effect provided by the metallic oxides, which increases binder stiffness and decreases its susceptibility to creep under cyclic loading at high temperatures. This behavior reinforces the potential of nanomaterials as effective modifiers for designing asphalt binders with enhanced performance, especially for applications under heavy traffic and hot climate conditions.

#### 4.2.3. Definition of the Optimized Content of ZnO + TiO_2_

To develop an asphalt mixture with optimized healing properties and superior rheological performance, an integrated and comparative analysis was conducted to determine the optimum incorporation content of nanomaterials (ZnO + TiO_2_) in the polymeric asphalt binder. The selection was based on thermal and mechanical performance criteria, including thermal conductivity (a key factor for the healing process), high-temperature performance grade (PGH), aging index, non-recoverable creep compliance, and apparent viscosity. As a result, the content of 8.5% ZnO + TiO_2_ was identified as the most suitable. At this concentration, the nanocomposite exhibited the following properties based on the trend line analysis:

- Thermal conductivity of 0.5 W/m·K, representing a 106.3% increase compared with the reference polymeric asphalt binder;
- Increase in PGH from 76-XX to 82-XX for both unaged and short-term aged (RTFOT) conditions;
- Mean reduction of 7.2% in the aging index (AI), indicating higher resistance to oxidation;
- Reduction in non-recoverable creep compliance (Jnr_3.2_): at 82 °C, the value decreased to 1.29 kPa^−1^, corresponding to a 24.9% reduction; at 76 °C, the value was 0.41 kPa^−1^, reflecting a 17.3% reduction;
- Apparent viscosity of 2.870 Pa·s, which is 4.3% below the SUPERPAVE limit of 3.0 Pa·s (135 °C), ensuring that binder workability is maintained during mixing and application.

These results indicate that the 8.5% ZnO + TiO_2_ content yields the most favorable combination of thermal and mechanical enhancements, suggesting that this condition may be suitable for application in asphalt mixtures. The following sections present and discuss the effects of this optimized incorporation on the behavior of the polymeric asphalt binder at intermediate temperatures. The analysis includes the rheological parameters of dynamic shear modulus (|G*|) and phase angle (δ), as well as fatigue resistance evaluated through the LAS test.

##### Phase Angle and Dynamic Shear Modulus Behavior at Intermediate Temperatures

Table 8 presents the influence of the combined addition of 8.5% ZnO + TiO_2_ to the polymeric asphalt binder, compared with the reference binder (N0—0% ZnO + TiO_2_), under short-term aged conditions (RTFOT). The analysis is based on rheological parameters obtained from the dynamic shear test, including the dynamic shear modulus (|G*|), phase angle (δ), storage modulus (G′, elastic component), and loss modulus (G″, viscous component). These parameters provide a comprehensive understanding of the viscoelastic behavior of the binder at intermediate temperatures, allowing the evaluation of the effects of nanometric modification on the mechanical characteristics of the material.

According to the data presented in Table 8, the incorporation of 8.5% ZnO + TiO_2_ into the polymeric asphalt binder resulted in consistent rheological behavior across the entire temperature range analyzed, from 5 °C to 35 °C. Considering the mean loading frequencies (0.1 Hz to 30 Hz), a generalized increase was observed in the dynamic shear modulus (|G*|), with variations ranging from 100.5% to 108.1% relative to the reference binder (without nanoparticle addition). The storage (G′) and loss (G″) moduli also increased, ranging from 101.3% to 109.3% for G′ and from 99.4% to 105.3% for G″. Regarding the phase angle (δ), the results demonstrated stable viscoelastic behavior, with minimal variations between +0.1% and −2.1%. This slight reduction in δ indicates a minor shift toward greater elastic dominance, yet without producing a substantial change in the ratio between the elastic and viscous responses. In summary, the results indicate that modification with 8.5% ZnO + TiO_2_ significantly enhances binder stiffness at intermediate temperatures without meaningfully altering the nature of its rheological behavior.

##### Linear Amplitude Sweep

Table 9 presents the rheological parameters derived from the Linear Amplitude Sweep (LAS) test for the reference asphalt binder (N0) and the binder modified with 8.5% ZnO + TiO_2_ (N_optimal_), both under short-term aged conditions (RTFOT). The fatigue life prediction equations, obtained based on viscoelastic models, were as follows: for the reference asphalt binder (N0), Nf = 10,350,246 γ^−5·21^, and for the optimized nanocomposite (N_optimal_), Nf = 9,226,490 γ^−5·24^.

Based on the data presented in Table 9, the incorporation of 8.5% ZnO + TiO_2_ into the reference asphalt binder resulted in changes in the fatigue model parameters derived from the LAS test. Parameter A, which represents the initial fatigue resistance under small deformations, decreased by 10.9% in the nanomodified binder (N_optimal_), suggesting a lower ability to delay the initiation of accumulated damage. Parameter B, associated with the sensitivity of the material to strain amplitude, increased by 0.6%, indicating that the nanomodified binder exhibits a greater dependence of fatigue life on the applied strain level. This behavior is corroborated by the number of cycles to failure (Nf), which was consistently lower for the binder containing nanomaterials across all tested strain amplitudes. The reductions ranged from 11.5% (for γ = 1.25%) to 17.8% (for γ = 15%), reflecting a decrease in durability under cyclic loading. Consequently, the fatigue factor (FF), used to estimate the overall binder performance, decreased by 1.0%, suggesting a negative impact of nanomaterial incorporation on damage tolerance. The reduction in fatigue life can be explained by the changes in viscoelastic properties previously identified at 20 °C, where a significant increase in the dynamic shear modulus (+102.9%) was observed, without a meaningful change in phase angle (−1.2%), indicating a stiffer behavior. Although this additional stiffness is beneficial for resisting deformation, it may compromise the ability of the binder to accommodate repeated stresses, particularly under fatigue conditions.

Despite the observed trends in the LAS test, it is important to note that this method is not yet widely consolidated within the scientific community as a definitive tool for predicting the contribution of the binder to the fatigue performance of asphalt mixtures, especially in systems modified with nanomaterials. The LAS test provides indirect estimates of fatigue resistance based solely on binder properties, disregarding essential aspects such as binder–aggregate interactions, gradation structure, composite matrix effects, and scale phenomena. These limitations make the results particularly sensitive to chemical and rheological modifications, as in the case of nanomodified binders. Therefore, the findings should be interpreted with caution and should not be used in isolation to predict the fatigue performance of asphalt mixtures.

### 4.3. Assessment of the Mechanical Performance of Asphalt Mixtures

With the optimized nanocomposite content established, the next step involved evaluating its behavior when applied in asphalt mixtures. In accordance with standard practice in binder-modification studies, only the optimum dosage was incorporated into the mixture, reflecting the condition previously identified as providing the most favorable combination of thermal and mechanical performance. Comprehensive experimental tests were conducted to investigate the effects of nanometric modification on the mechanical performance of the mixture. The procedures included: the permanent deformation test to assess resistance to permanent deformation; the characterization of viscoelastic behavior at intermediate temperatures; the fatigue test to estimate durability under repeated loading; the healing protocol to evaluate self-healing ability; and the assessment of the internal heating rate of the asphalt mixtures under microwave radiation

#### 4.3.1. Permanent Deformation Resistance

**Figure 19:**
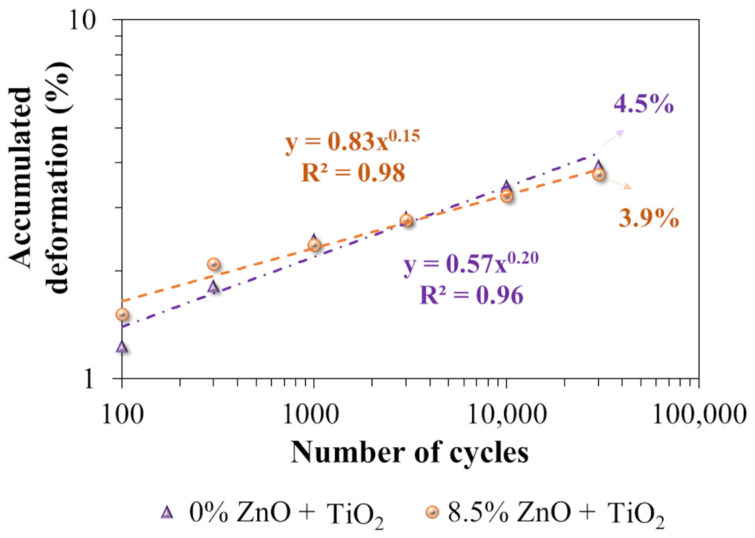
Accumulated deformation of reference (M1—0% ZnO + TiO_2_) and nanomodified (M2—8.5% ZnO + TiO_2_) asphalt mixtures in relation to the number of applied cycles.

The rutting performance was analyzed using the French Orniéreur traffic simulator, testing two specimens of the reference asphalt mixture (M1—0% ZnO + TiO_2_) and two specimens of the nanomodified asphalt mixture (M2—8.5% ZnO + TiO_2_). In Figure 19, the values represent the mean results of the tests, while the trend line and characteristic equation were derived from these data.

The results presented in Figure 19 illustrate the accumulated deformation (%) as a function of the number of loading cycles for two asphalt mixture formulations: the reference mixture (M1, with 0% ZnO + TiO_2_) and the nanomodified mixture (M2, with 8.5% ZnO + TiO_2_). Data analysis shows that, during the first four loading intervals (100, 300, 1000, and 3000 cycles), the reference mixture exhibited lower rutting levels. However, after 10,000 cycles, this trend reversed, and the mixture containing nanomaterials began to display lower accumulated deformation values. At the end of 30,000 cycles, the nanomodified mixture (M2) exhibited a rut depth of 3.9%, which was lower than the 4.5% observed for the conventional mixture (M1). This inversion over time indicates that, while the reference mixture shows better initial resistance to deformation, the nanomodified mixture presents a lower rate of permanent deformation progression.

The higher coefficient of M2 (0.83) explains its slightly greater initial deformation; however, its smaller exponent (0.15 < 0.20) indicates a lower rate of deformation growth with cycles, which explains why M2 outperforms M1 in the long term. Rheologically, although M2 exhibits higher |G*| and lower δ (greater stiffness and elasticity), as previously reported in this study, its stiffer internal network accumulates stress during the first loading cycles. As the load repetitions progress, this structure restricts creep and rut development, resulting in lower total deformation at the end of the test.

The superior performance of mixture M2 can be attributed to the improved rheological properties of the nanomodified binder, as previously demonstrated in this study. Notably, the increased apparent viscosity, higher |G*|/sin δ parameter values, and reduced non-recoverable deformation observed in MSCR tests all indicate greater binder stiffness and creep resistance, factors that directly contribute to mitigating plastic deformation in asphalt mixtures under repeated loading.

These findings are consistent with the literature. Kamboozia et al. [24], when analyzing porous mixtures with different ZnO contents, observed a progressive reduction in permanent deformation with increasing nanoparticle concentration. Similarly, Sadeghnejad and Shafabakhsh [21] reported significant improvements in rutting resistance in SMA-type mixtures modified with nano-TiO_2_, attributing this behavior to enhanced interaction between asphalt particles and nanomaterials, which increases internal cohesion and resistance to deformation.

Therefore, the results indicate that the combined addition of 8.5% ZnO + TiO_2_ to the polymeric binder can improve the permanent deformation resistance of the mixture, particularly under heavy traffic and high-temperature conditions, underscoring its potential for high-performance pavement applications.

#### 4.3.2. Rheological Behavior at Intermediate Temperatures

Table 10 presents the results obtained for the phase angle and dynamic modulus at intermediate temperatures (30 °C to 0 °C) for the reference asphalt mixture (M1—0% ZnO + TiO_2_) and the nanomodified asphalt mixture (M2—8.5% ZnO + TiO_2_). The table reports the mean values, considering all loading frequencies (0.1 Hz to 20 Hz), for the dynamic modulus (|E*|), phase angle (δ), and the elastic (E_1_) and viscous (E_2_) components.

The data presented in Table 10 demonstrate that the incorporation of 8.5% ZnO + TiO_2_ into the polymeric binder produced a consistent increase in the dynamic modulus (|E*|) of the asphalt mixture throughout the entire temperature range tested (0 °C to 30 °C). The effect was most pronounced at higher temperatures, particularly at 30 °C, where the greatest increase in |E*| (+31.0%) was recorded compared to the reference mixture. The magnitude of this improvement gradually decreased with lower temperatures, showing increments of +21.1% (25 °C), +12.9% (20 °C), +9.5% (15 °C), +5.8% (10 °C), +3.8% (5 °C), and +4.5% (0 °C). This increase in |E*| reflects greater structural stiffness in the nanomodified mixture, attributed to the reinforcing effect of the metallic oxides on the matrix. Such behavior is particularly beneficial for reducing accumulated deformation under repeated loading, as previously observed in the permanent deformation test. Furthermore, these results are consistent with the rheological data of the modified binders obtained from the PGH and MSCR tests, in which the binder containing 8.5% ZnO + TiO_2_ also exhibited superior performance.

The phase angle (δ) (Table 10) showed reductions across the entire temperature range analyzed, varying between −4.6% (30 °C) and −10.3% (10 °C), indicating a shift toward more elastic behavior in the mixture. Reductions of −8.8% (5 °C) and −9.0% (0 °C) further confirm this trend at lower temperatures. The decrease in δ is associated with the predominance of the storage modulus (E_1_) over the loss modulus (E_2_), indicating increased resistance to viscoelastic deformation.

Overall, the results show that modification with metallic oxides promotes a rheological reinforcement, characterized by increased stiffness and greater elastic dominance, features that contribute to superior asphalt mixture performance against permanent deformation, fatigue, and thermal variations.

#### 4.3.3. Fatigue Resistance and Healing Capacity

**Figure 20:**
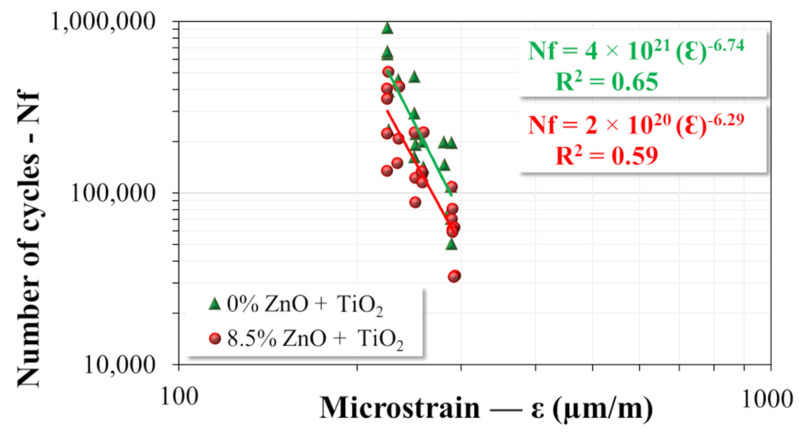
Fatigue curves: reference (M1—0% ZnO + TiO_2_) and nanomodified (M2—8.5% ZnO + TiO_2_) asphalt mixture.

The fatigue performance of the asphalt mixtures was evaluated using the four-point bending beam test. The fatigue test was initially conducted until failure for both mixtures (reference—M1 and nanomodified—M2). Subsequently, the specimens used in the first fatigue test were immediately subjected to the healing protocol. Afterward, a new fatigue test was performed to assess the residual life of the samples following the healing procedure. The results obtained from the first fatigue test are presented in Figure 20, Table 11 and Table 12.

As shown in Figure 20 and Table 11, the reference asphalt mixture exhibited superior performance compared with the nanomodified mixture in terms of fatigue resistance. For the same strain level, the reference mixture withstood a higher number of cycles to failure (Nf), indicating a greater ability to resist fatigue damage in laboratory conditions. This result suggests that the modification with ZnO and TiO_2_ nanoparticles, at a concentration of 8.5%, reduced the fatigue resistance of the asphalt composite. The analysis of parameter b in the fatigue life equations, presented in Figure 20, revealed that the nanomodified mixture exhibited 6.7% lower strain susceptibility (b = −6.29) compared with the reference mixture (b = −6.74).

Table 11 presents the mean values of the initial dynamic modulus, measured at the 100th loading cycle. The nanomodified mixture (M2) showed a mean modulus of 7328 MPa (SD = 526 MPa), representing a 4.1% increase compared with the reference mixture (M1), which had a mean modulus of 7043 MPa (SD = 591 MPa). This increase in stiffness was corroborated by complementary analyses, such as the dynamic shear modulus of the nanomodified binder (N_optima_l) and the dynamic modulus of the M2 mixture itself, both evaluated at 20 °C. However, the higher stiffness observed may be associated with the reduced fatigue resistance, since stiffer materials tend to have lower energy dissipation capacity and, consequently, greater susceptibility to cracking. This trend was also confirmed by the LAS test performed at 20 °C on the nanomodified binder, which demonstrated lower fatigue resistance.

On the other hand, the nanomodified asphalt mixture (M2), due to its higher stiffness, tends to experience lower levels of strain under loading in service conditions when compared to the reference mixture (M1). In laboratory tests conducted under controlled-strain loading, stiffer materials generally reach failure in fewer cycles. However, this response does not necessarily reflect their behavior in the field, where higher stiffness tends to limit the strains experienced by the material. Therefore, the lower number of cycles required to reach the same controlled strain does not, by itself, indicate reduced fatigue resistance, but rather reflects the change in the mechanical response of the mixture resulting from the increased stiffness.

Additionally, the fatigue factor of the mixture (FFM), presented in Table 12, indicates that the nanomodified mixture exhibited an FFM 4.4% lower than that of the reference asphalt mixture. This result reinforces that, at the concentration adopted, the incorporation of nanomaterials did not provide improvements in fatigue resistance at the same deformation amplitude. The results obtained contrast with those reported by Sadeghnejad and Shafabakhsh [21], who observed an increase in fatigue life with the incorporation of up to 1.2% TiO_2_ in Stone Mastic Asphalt (SMA) mixtures evaluated under indirect tensile tests. Similarly, Mousavi Rad et al. [23] observed improved fatigue performance with up to 8% ZnO in porous mixtures using four-point bending tests. This divergence can be attributed to three main factors: (i) differences in the binders used (conventional in previous studies versus SBS-modified in this study); (ii) variations in testing methodology, including the use of indirect tensile tests in Sadeghnejad and Shafabakhsh [21] and higher strain amplitudes (500 and 700 microstrains) used by Mousavi Rad et al. [23]; and (iii) the higher nanoparticle concentration adopted in this study (8.5%) compared with the maximum levels used in the previous studies (1.2% and 8%, respectively).

Regarding the healing capacity, Table 13 presents the results of the second fatigue test conducted for both asphalt mixtures (M1 and M2). The table also shows the calculated healing percentages, which allow for the evaluation of the effectiveness of the damage recovery process induced by fatigue in each mixture.

The results in Table 13 indicate that the nanomodified asphalt mixture exhibited superior performance compared with the reference mixture in terms of mean healing capacity. The mean healing percentage obtained for the modified mixture was 23.5%, compared to 15.5% for the reference mixture, representing a 51.6% increase attributed to the presence of nanomaterials. When the healing values are converted into their normalized form (Equation (9)), the results are 23.2 × 10^−8^ [1/(J/m^3^)] for the nanomodified mixture and 15.4 × 10^−8^ [1/(J/m^3^)] for the reference mixture. The improvement in healing under this metric corresponds to a 50.7% increase, reinforcing the evidence that the combined addition of ZnO and TiO_2_ has a positive impact on the healing capacity of the asphalt mixture.

The strain-range analysis reveals that this effect is more pronounced at lower microstrain levels. Specifically, within the range of 225 µm/m to 236 µm/m, the nanomodified mixture achieved a healing rate of 21.7%, while the reference mixture reached only 5.5%, representing a substantial increase of 294.6%. According to Nascimento [116], microstrain levels between 100 µm/m and 200 µm/m are commonly observed at the base of asphalt layers in Brazilian pavements, based on typical traffic-load simulations. Therefore, the data suggest a superior performance of the nanomodified mixture under service-level strain conditions.

#### 4.3.4. Assessment of the Internal Heating Rate of Asphalt Mixtures Under Microwave Radiation

**Figure 21:**
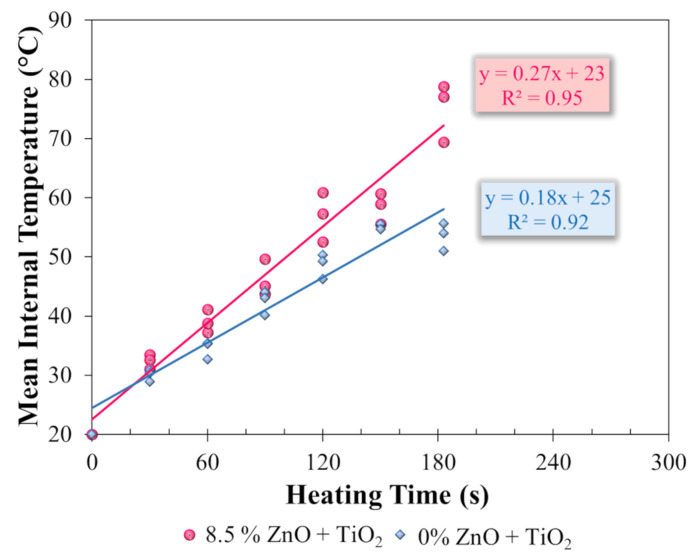
Mean internal temperature as a function of microwave heating time of asphalt mixtures.

Following the healing procedure, the mean internal temperature of the asphalt mixtures M1 (0% ZnO + TiO_2_) and M2 (8.5% ZnO + TiO_2_) was measured as a function of exposure time to microwave heating, as illustrated in Figure 21. The purpose of this stage was to verify the hypothesis that the incorporation of nanomaterials would increase the internal heating rate of the mixture, thereby enhancing the healing process.

According to Figure 21, the reference asphalt mixture (M1—0% ZnO + TiO_2_) exhibited an internal temperature increase rate of 0.18 °C/s, whereas the nanomodified mixture (M2—8.5% ZnO + TiO_2_) reached a rate of 0.27 °C/s. This difference represents a 50.0% increase in the heating rate compared with the conventional mixture, demonstrating the direct impact of nanomaterials on the thermal response of the composite. Considering the total exposure time of 140 s to microwave heating, the mean internal temperature reached 50.2 °C for M1 and 60.8 °C for M2, corresponding to an increase of approximately 21.2% due to the addition of metallic oxides.

This behavior highlights the enhanced thermal efficiency of the nanomodified mixture, resulting from the greater absorption and heat conduction promoted by ZnO and TiO_2_ nanoparticles. However, because both ZnO and TiO_2_ are semiconductor oxides with microwave–dielectric interaction [117,118,119,120], the observed temperature rise cannot be attributed solely to improved thermal conductivity. Instead, it likely results from the combined effect of heat conduction and microwave-induced dielectric heating, which could not be isolated under the conditions of this study.

Therefore, the superior healing capacity observed in the M2 mixture can be attributed, at least in part, to the faster and more intense increase in internal temperature during the heating process, which facilitates the mobilization of the polymeric asphalt binder and promotes the recovery of the fatigue-damaged structure. Nevertheless, the contribution of dielectric heating must be acknowledged as a potentially significant mechanism that remains unquantified and constitutes a major limitation of the present interpretation.

## 5. Conclusions

This study aimed to optimize the combined incorporation of nano-ZnO and nano-TiO_2_ (50/50 wt.%) into a polymer-modified asphalt binder and to evaluate the effects of this nanocomposite on the mechanical performance and healing capacity of asphalt mixtures. The main conclusions are as follows:

- The combined addition of ZnO and TiO_2_ at contents ranging from 2 to 12 wt.% (in 2% increments) enhanced the high-temperature performance of the polymer-modified binder, increasing its thermal conductivity and resistance to permanent deformation.
- A dosage of 8.5 wt.% provided the most favorable combination of thermal and rheological properties, resulting in higher thermal conductivity, improved high-temperature stability, and reduced rutting susceptibility, while maintaining workability within the SUPERPAVE limit (≤3.0 Pa·s at 135 °C).
- The asphalt mixture produced with the binder modified with 8.5 wt.% ZnO + TiO_2_ exhibited higher stiffness at intermediate temperatures, a 50.7% increase in normalized healing efficiency, and 13.3% greater resistance to permanent deformation, demonstrating the contribution of the nanoparticles to mechanical reinforcement and structural recovery.
- The improvement in healing capacity was associated with a 50.0% increase in the internal heating rate, which accelerated binder mobilization, increased molecular mobility, and facilitated crack closure and the reconstruction of fatigue-damaged microstructures.
- Regarding fatigue performance under the same imposed microstrain levels, the incorporation of ZnO + TiO_2_ into the asphalt mixture resulted in a 4.4% reduction in the fatigue factor. This behavior is attributed to the increased stiffness provided by the nanomaterials, which reduces the ability of the mixture to deform under loading.
- Overall, the ZnO + TiO_2_ combination enhances the functional performance of asphalt materials, adding benefits in resistance to permanent deformation and healing capacity in addition to the well-known photocatalytic potential reported in the literature, making this nanocomposite a promising alternative for pavements subjected to high temperatures and heavy traffic.

Although this study satisfactorily addressed the behavior of the materials at high and intermediate temperatures, several aspects warrant further investigation. Future research should examine the performance of the nanocomposites and mixtures at low temperatures, evaluate the economic feasibility of incorporating nanomaterials, conduct comparative studies between microwave heating and equivalent conventional heating to isolate thermal-conductivity-related effects from dielectric heating, and investigate the influence of different ZnO:TiO_2_ ratios to determine whether asymmetric formulations can further enhance microwave-heating efficiency and the healing performance of the asphalt mixture. Additional efforts should also focus on assessing the potential release of nanoparticles during production and service and developing methods for directly characterizing nanoparticle dispersion in asphalt matrices. Together, these investigations would provide a more comprehensive understanding of the effects of ZnO and TiO_2_ on asphalt mixture performance and durability.

## Figures and Tables

**Table 1:** Properties of mineral aggregates [37].

Properties	Test Method	Results
Hardness (Los Angeles abrasion)	ASTM C131 [38]	18.64%
Absorption of coarse aggregate	ASTM C127 [39]	1.43%
Angularity of coarse aggregate	ASTM D5821 [40]	100%
Angularity of fine aggregate	ASTM C1252 [41]	52.36%
Clay content (sand equivalent)	AASHTO T 176 [42]	71.95%
Bulk specific gravity (Gsb) of coarse aggregate	ASTM C127 [39]	2.592 g/cm^3^
Apparent specific gravity (Gsa) of coarse aggregate	ASTM C127 [39]	2.648 g/cm^3^
Deleterious material	AASHTO T 112 [43]	0.00%
Soundness	ASTM C88 [44]	1.59%

**Table 2:** Results of characterization tests of polymer-modified asphalt binder [45].

Properties	Test Method	Results
Phase separation (∆PA)	NBR 15166 [46]	0.3 °C
Density, 25 °C	NBR 6296 [47]	1.009
Penetration, 100 g, 5 s, 25 °C	NBR 6576 [48]	48 (0.1 mm)
Softening point	NBR 6560 [49]	68 °C
Flash and fire points	NBR 11341 [50]	254 °C
Elastic recovery, 20 cm, 25 °C	NBR 15086 [51]	90.0%
Solubility in trichloroethylene	NBR 14855 [52]	99.9% (mass)

**Table 3:** Typical properties of D1101 A copolymer [53].

Property	Test Method	Typical Value
Specific gravity	ISO 2781 [54]	0.94
Bulk density	ASTM D1895 method B [55]	0.4 kg/dm^3^
Melt flow rate, 200 °C/5 kg	ISO 1133 [56]	<1 g/10 min
Tensile strength	ISO 37 [57]	33 MPa
300% modulus	ISO 37 [57]	2.9 MPa
Elongation at break	ISO 37 [57]	880%
Hardness, shore A (30 s)	ISO 868 [58]	72

**Table 4:** Properties of ZnO and TiO_2_ nanoparticles [59,60].

Properties	ZnO	TiO_2_
Purity	>99%	>99%
Mean particle size	20 nm	10 nm
Morphology of particles	Spherical	Ellipsoidal/spherical
Specific surface area	≥40 m^2^/g	≥60 m^2^/g
Bulk density	0.1–0.2 g/cm^3^	0.2–0.3 g/cm^3^
pH value	6.5–7.5	5.5–7.0
Loss on drying (110 °C/2 h)	≤1.0 wt.%	≤2.0 wt.%
Loss on calcination (850 °C/2 h)	≤3.0 wt.%	≤5.0 wt.%

**Table 5:** Produced asphalt nanocomposites.

Number of Produced Nanocomposites	Incorporation (by Binder Weight)		Produced Nanocomposite
	Nanomaterial		
	ZnO	TiO_2_	
N0 (reference)	0%	0%	0% (ZnO + TiO_2_)
N2	1%	1%	1% (ZnO + TiO_2_)
N4	2%	2%	2% (ZnO + TiO_2_)
N6	3%	3%	3% (ZnO + TiO_2_)
N8	4%	4%	4% (ZnO + TiO_2_)
N10	5%	5%	5% (ZnO + TiO_2_)
N12	6%	6%	6% (ZnO + TiO_2_)

**Table 6:** Granulometric composition of asphalt mixtures [82]. “No.” indicates sieve number and “in.” denotes aperture size in inches.

Aggregate Fraction	Sieves (ASTM Series)	% Retained
Coarse aggregates	1/2 in.	22.5
	3/8 in.	16.2
	No. 4	18.0
Fine aggregates	No. 10	19.0
	No. 16	6.9
	No. 30	4.8
	No. 50	2.8
	No. 100	2.2
	No. 200	2.2
Filler	Filler	5.4

**Table 7:** Games-Howell post hoc test.

		N0	N2	N4	N6	N8	N10	N12
N0	Mean difference	-	−0.0683	−0.0955	−0.135	−0.1895	−0.2698	−0.3163
	*p*-value	-	<0.001	<0.001	<0.001	<0.001	<0.001	<0.001
N2	Mean difference		-	−0.0272	−0.0667	−0.1212	−0.2015	−0.248
	*p*-value		-	<0.001	<0.001	<0.001	<0.001	<0.001
N4	Mean difference			-	−0.0395	−0.094	−0.1743	−0.2208
	*p*-value			-	0.003	<0.001	<0.001	<0.001
N6	Mean difference				-	−0.0545	−0.1348	−0.1813
	*p*-value				-	<0.001	<0.001	<0.001
N8	Mean difference					-	−0.0803	−0.1268
	*p*-value					-	<0.001	<0.001
N10	Mean difference						-	−0.0465
	*p*-value						-	<0.001
N12	Mean difference							-
	*p*-value							-

**Table 8:** Results of the dynamic shear modulus (|G*|), phase angle (δ), and elastic (G′) and viscous (G″) components of the reference (0% ZnO + TiO_2_) and nanomodified (8.5% ZnO + TiO_2_) asphalt mixtures, evaluated at different temperatures (35 °C to 5 °C) considering the mean across all loading frequencies (0.1 Hz to 30 Hz).

Temperature	Parameter	Mean (0.1 Hz–30 Hz)		% Variation
		0% ZnO + TiO_2_ (N0)	8.5% ZnO + TiO_2_ (N_optimal_)	
35 °C	|G*| (kPa)	394	802	103.4%
	δ (°)	64.1	64.2	0.1%
	G′ (kPa)	178	362	103.1%
	G″ (kPa)	352	715	103.3%
30 °C	|G*| (kPa)	985	2019	105.0%
	δ (°)	61.6	61.3	−0.4%
	G′ (kPa)	512	1054	106.0%
	G″ (kPa)	840	1717	104.3%
25 °C	|G*| (kPa)	2645	5419	104.9%
	δ (°)	55.9	55.5	−0.8%
	G′ (kPa)	1630	3377	107.2%
	G″ (kPa)	2075	4225	103.6%
20 °C	|G*| (kPa)	6332	12,846	102.9%
	δ (°)	49.2	48.6	−1.2%
	G′ (kPa)	4463	9116	104.2%
	G″ (kPa)	4467	9001	101.5%
15 °C	|G*| (kPa)	13,510	27,091	100.5%
	δ (°)	42.5	41.9	−1.3%
	G′ (kPa)	10,470	21,075	101.3%
	G″ (kPa)	8482	16,915	99.4%
10 °C	|G*| (kPa)	26,630	55,413	108.1%
	δ (°)	35.8	35.1	−2.1%
	G′ (kPa)	22,303	46,685	109.3%
	G″ (kPa)	14,425	29,615	105.3%
5 °C	|G*| (kPa)	48,232	99,115	105.5%
	δ (°)	30.4	29.9	−1.7%
	G′ (kPa)	42,462	87,556	106.2%
	G″ (kPa)	22,644	46,015	103.2%

**Table 9:** LAS test parameters calculated with the “AASHTO T 391-20—Version 1.59” [80] spreadsheet (MARC website) for the test temperature of 20 °C. Nf: number of cycles until rupture; FF: asphalt binder fatigue factor; γ: applied shear strain; SD: standard deviation; and CV: coefficient of variation.

Parameter	N0 (0% ZnO + TiO_2_)			N_optimal_ (8.5% ZnO + TiO_2_)			% Variation
	Mean	SD	CV	Mean	SD	CV	
A	10,350,246	1,753,858	17.0%	9,226,490	1,399,268	15.2%	−10.9%
B	5.21	0.01	0.1%	5.24	0.02	0.4%	0.6%
Nf (γ: 1.25%)	3,236,134	544,325	16.8%	2,865,510	436,161	15.2%	−11.5%
Nf (γ: 2.50%)	87,417	14	16.4%	75,817	11,720	15.5%	−13.3%
Nf (γ: 5.00%)	2361	379	16.1%	2006	317	15.8%	−15.0%
Nf (γ: 7.50%)	286	45	15.9%	240	38	16.0%	−16.1%
Nf (γ: 10.00%)	64	10	15.7%	53	9	16.2%	−16.8%
Nf (γ: 12.50%)	20	3	15.6%	16	3	16.4%	−17.3%
Nf (γ: 15.00%)	8	1	15.5%	6	1	16.5%	−17.8%
FF (γ: 1.25–2.50%)	1.72	0.02	1.3%	1.71	0.02	1.1%	−1.0%

**Table 10:** Results of the dynamic modulus (|E*|), phase angle (δ), and elastic (E_1_) and viscous (E_2_) components of the reference (M1—0% ZnO + TiO_2_) and nanomodified (M2—8.5% ZnO + TiO_2_) asphalt mixtures, evaluated at different temperatures (30 °C to 0 °C) considering the mean across all loading frequencies (0.1 Hz to 20 Hz).

Temperature	Parameter	Mean (0.1 Hz–20 Hz)		% Variation
		0% ZnO + TiO_2_ (M1)	8.5% ZnO + TiO_2_ (M2)	
30 °C	|E*| (kPa)	1,536,667	2,012,889	31.0%
	δ (°)	42.1	40.1	−4.6%
	E_2_ (kPa)	1,004,944	1,230,108	22.4%
	E_1_ (kPa)	1,160,868	1,588,547	36.8%
25 °C	|E*| (kPa)	2,774,778	3,359,333	21.1%
	δ (°)	36.6	34.5	−5.9%
	E_2_ (kPa)	1,531,254	1,711,363	11.8%
	E_1_ (kPa)	2,304,012	2,873,802	24.7%
20 °C	|E*| (kPa)	5,293,667	5,975,778	12.9%
	δ (°)	27.4	25.2	−7.9%
	E_2_ (kPa)	2,171,383	2,280,832	5.0%
	E_1_ (kPa)	4,802,030	5,495,797	14.5%
15 °C	|E*| (kPa)	8,268,111	9,056,778	9.5%
	δ (°)	19.7	17.9	−9.2%
	E_2_ (kPa)	2,488,840	2,502,437	0.6%
	E_1_ (kPa)	7,852,371	8,673,551	10.5%
10 °C	|E*| (kPa)	11,605,000	12,281,333	5.8%
	δ (°)	14.0	12.5	−10.3%
	E_2_ (kPa)	2,580,523	2,463,172	−4.6%
	E_1_ (kPa)	11,291,725	12,008,692	6.4%
5 °C	|E*| (kPa)	15,103,444	15,672,778	3.8%
	δ (°)	9.6	8.7	−8.8%
	E_2_ (kPa)	2,350,058	2,240,258	−4.7%
	E_1_ (kPa)	14,903,350	15,498,457	4.0%
0 °C	|E*| (kPa)	18,560,556	19,388,889	4.5%
	δ (°)	6.4	5.9	−9.0%
	E_2_ (kPa)	1,970,855	1,901,843	−3.5%
	E_1_ (kPa)	18,444,338	19,288,878	4.6%

**Table 11:** Results of the test specimens of the reference (M1) and nanomodified (M2) asphalt mixture tested for fatigue in the 4-point bending equipment. ε (µm/m): effective microstrain applied to the specimen; |E*| initial: initial dynamic modulus measured at the 100th loading cycle; Nf: number of applications of the request until the initial dynamic modulus is reduced by 50% and SD: standard deviation.

Reference Asphalt Mixture (M1)			Nanomodified Asphalt Mixture (M2)		
ε (µm/m)	|E*| Initial (MPa)	Nf	ε (µm/m)	|E*| Initial (MPa)	Nf
281	7741	145,611	289	7401	108,932
288	7983	71,034	289	7236	70,574
289	7163	50,745	292	7142	63,355
288	7980	109,200	293	7252	32,980
289	7585	196,106	290	7774	80,782
280	6893	197,173	290	8163	61,364
287	7933	79,516	291	7236	32,584
251	6226	191,576	290	7716	59,148
251	6610	220,823	251	6270	122,997
250	7318	290,632	250	6285	226,283
250	6209	476,063	251	7115	88,356
250	6375	161,744	258	7987	128,532
258	6769	199,664	257	6836	133,059
259	6911	141,592	258	8212	115,448
225	7257	641,263	259	7043	224,878
225	6444	914,578	225	7171	405,659
225	6568	665,988	225	6526	134,329
226	6769	233,245	226	7647	505,834
226	6613	391,755	225	7328	222,345
235	7503	455,050	235	7647	206,528
			225	7804	354,816
			236	7571	414,825
			234	7181	149,794
Mean	7043		Mean	7328	
SD	591		SD	526	

**Table 12:** Fatigue factor of reference and nanomodified asphalt mixtures.

Asphalt Mixture	0% ZnO + TiO_2_ (M1)	8.5% ZnO + TiO_2_ (M2)
Equation	Nf = 4 × 10^21^ (ε)^−6.74^	Nf = 2 × 10^20^ (ε)^−6.29^
Nf_100_	123,448,265	49,674,175
Nf_250_	256,667	155,987
FFM	2.70	2.58

**Table 13:** Mean percentage of healing of reference (M1) and nanomodified (M2) asphalt mixtures. H: Healing.

Reference Asphalt Mixture (M1)				Nanomodified Asphalt Mixture (M2)			
ε (µm/m)	Number of Cycles (Nf)		%H	ε (µm/m)	Number of Cycles (Nf)		%H
	1st Fatigue Test	2st Fatigue Test			1st Fatigue Test	2st Fatigue Test	
281	145,611	31,636	21.7	289	108,932	20,946	19.2
288	71,034	12,749	18.0	289	70,574	11,536	16.4
289	50,745	12,627	24.9	292	63,355	23,495	37.1
288	109,200	23,657	21.7	293	32,980	8849	26.8
289	196,106	50,030	25.5	290	80,782	14,265	17.7
280	197,173	32,651	16.6	290	61,364	11,400	18.6
287	79,516	22,042	27.7	291	32,584	10,497	32.2
251	191,576	18,107	9.5	290	59,148	18,761	31.7
251	220,823	57,319	26.0	251	122,997	29,199	23.7
250	290,632	53,256	18.3	250	226,283	52,831	23.4
250	476,063	69,435	14.6	251	88,356	26,902	30.5
250	161,744	8323	5.2	258	128,532	21,427	16.7
258	199,664	58,945	29.5	257	133,059	43,623	32.8
259	141,592	25,928	18.3	258	115,448	24,221	21.0
225	641,263	18,993	3.0	259	224,878	42,732	19.0
225	914,578	11,938	1.3	225	405,659	97,278	24.0
225	665,988	30,185	4.5	225	134,329	37,328	27.8
226	233,245	16,008	6.9	226	505,834	115,000	22.7
226	391,755	31,750	8.1	225	222,345	44,690	20.1
235	455,050	42,625	9.4	235	206,528	35,318	17.1
				225	354,816	50,815	14.3
				236	414,825	103,724	25.0
				234	149,794	33,563	22.4
%H Mean			15.5	%H Mean			23.5

## Data Availability

The original contributions presented in this study are included in the article. Further inquiries can be directed to the corresponding author.
